# Integrating oxygen-boosted sonodynamic therapy and ferroptosis *via* engineered exosomes for effective cancer treatment

**DOI:** 10.7150/thno.102977

**Published:** 2025-01-01

**Authors:** Mingbo Wu, Zhanlin Zhang, Dong Li, Xiaomiao Ruan, Jingwen Yang, Siyi Chen, Xin Li, Wenwu Ling

**Affiliations:** 1 Department of Medical Ultrasound, West China Hospital, Sichuan University, Chengdu 610041, P.R. China.; 2 Department of Oncology, The General Hospital of Western Theater Command, Chengdu610083, P.R. China.; 3 School of Bioscience and Technology, Chengdu Medical College, Chengdu 610500, P.R. China.

**Keywords:** Engineered exosome, Sonodynamic therapy, Ferroptosis, Tumor microenvironment, Active penetration

## Abstract

**Rationale:** Ferroptosis and sonodynamic therapy (SDT) are both promising therapeutic modalities, but their clinical application remains challenging due to the hypoxic tumor microenvironment and limited supply of polyunsaturated fatty acids. Developing an agent with oxygen-enhanced SDT and increased ferroptosis sensitivity is crucial for advancing tumor therapy.

**Methods:** In this study, catalase (Cat) and Acyl-CoA synthetase long-chain family member 4 (ACSL4) highly expressed 4T1 cells were constructed *via* lentivirus transfection. Cat and ACSL4 enriched exosomes (EXO@CA) were then extracted and loaded with the sonosensitizer tetrakis (4-carboxyphenyl) porphyrin (TCPP) through electroporation to create engineered exosomes (EXO@CAT). We evaluated the ability of EXO@CAT to generate oxygen in a hydrogen peroxide environment and investigated its effect on motion profiles and permeability of EXO@CAT. The* in vitro* antitumor activity was assessed *via* cytotoxicity, ROS levels, live/dead staining, and apoptosis, with ferroptosis biomarkers confirming ferroptosis activation. We also evaluated the *in vivo* anticancer efficacy of EXO@CAT by tumor growth analysis and histological and immunohistochemical staining in mouse models bearing breast tumor.

**Results:** EXO@CAT harnesses ultrasound stimulation to facilitate oxygen-enriched SDT, demonstrating significant capacity for singlet oxygen (^1^O_2_) generating, which promotes the accumulation of lipid peroxidation (LPO), ultimately leading to the induction of ferroptosis. Concurrently, ACSL4 released from EXO@CAT also increases LPO accumulation by modifying cellular lipid composition, thereby enhancing cellular sensitivity to ferroptosis. Moreover, both *in vitro* and *in vivo* experiments demonstrate that the homologous targeting ability of EXO@CAT enables its efficient accumulation in tumor tissues, and the oxygen generation catalyzed by Cat not only alleviates tumor hypoxia but also facilitates the penetration of EXO@CAT into deeper layers of tumor tissue.

**Conclusions:** EXO@CAT combines endogenous proteins, which are prone to inactivation, with an exogenous sonosensitizer, allowing synergistic anticancer treatment of both ferroptosis and SDT with improved efficacy.

## Introduction

Excessive accumulation of reactive oxygen species (ROS) has been identified as an effective therapeutic strategy for tumors, as ROS can disrupt intracellular redox homeostasis, leading to irreversible damage to tumor cell organelles and essential biomolecules 1. Among various ROS-based cancer therapeutic modalities, ferroptosis has garnered significant attention due to its outstanding tumor-selective and non-invasive properties, demonstrating promise in the treatment of a variety of cancers, including lung cancer, ovarian cancer, breast cancer, and leukemia 2. Ferroptosis is generally understood to be triggered by aberrant intracellular ROS production, which results in a massive accumulation of lipid peroxidation (LPO) on the cell membrane. This process is typically associated with the inhibition of glutathione peroxidation 4 (GPX4) activity, which is mediated by the depletion of glutathione (GSH) 3, 4. Excessive LPO of unsaturated fatty acids on the cell membrane is considered a critical hallmark of ferroptosis 5. Unsurprisingly, ferroptosis treatment involves important physiological processes such as the regulation of cellular redox homeostasis and lipid metabolism; thus, enhancing ROS production and promoting LPO serve as sensitizing factors for the activation of ferroptosis 6, 7. Based on this understanding, various non-invasive ROS therapeutic modalities, such as chemodynamic therapy (CDT) 8, photodynamic therapy (PDT) 9, and sonodynamic therapy (SDT) 10 have been developed to enhance the efficacy of ferroptosis in treating different types of cancer.

Compared to PDT, SDT is particularly advantageous in the clinical treatment of deep tumors due to its superior tissue penetration depth and minimal side effects. SDT relies the activation of sonosensitizers to generate highly cytotoxic ROS, predominantly singlet oxygen (^1^O_2_), under low-intensity ultrasound (US) stimulation, ultimately induce tumor cell death 11. The conventional US-boosted ferroptosis nano formulation is composed of sonosensitizers and ferroptosis activator, which are released in the tumor microenvironment. The ^1^O_2_ produced through US-activated SDT not only induces apoptotic cell death, but also leads to a significant accumulation of LPO, thereby facilitating ferroptosis 3, 10. Despite numerous investigations confirming that ROS generated by SDT can irreversibly induce LPO accumulation and activate ferroptosis, the hypoxic tumor microenvironment (TME) limits the therapeutic efficacy of SDT. The rapid oxygen depletion during SDT can exacerbate hypoxia, further inhibiting SDT efficiency 12. Therefore, an effective method to alleviate tumor hypoxia involves delivering catalase (Cat) to tumor cells to catalyze the intracellular generation of oxygen from high levels of endogenous hydrogen peroxide (H_2_O_2_) 13, 14. This approach is important for improving the synergistic therapeutic effects of SDT and ferroptosis. However, the challenges related to the retention of activity, encapsulation efficiency, and delivery effects of Cat as an active protein remain significant obstacles to its application in overcoming hypoxic TME.

Ferroptosis-related LPO is significantly influenced by the relative abundance of polyunsaturated fatty acids (PUFAs) in biomembranes and the enzymes involved in their synthesis and peroxidation 15. Consequently, in addition to the direct introduction of exogenous lipids, enhancing the catalytic activity of LPO-mediated enzymes or increasing the synthesis of PUFA can also serve as effective strategies to promote susceptibility to ferroptosis. Acyl-CoA synthetase long-chain family member 4 (ACSL4) is an important metabolic isozyme responsible for polyunsaturated fatty acids (PUFAs), which can convert free PUFAs into membrane phospholipids *via* esterification reactions for LPO 16. Recent studies have shown that ACSL4 is an essential enzyme for the activation of ferroptosis, whereby its elevated expression increases cellular susceptibility to ferroptosis 17. Zheng *et al.* prepared a ferroptosis inducer, PGHM-R-Fe, which utilizes protein-based nanocomplexes (PGHM-R) as the stabilizer and carrier of Fe^2+^. When being internalized by tumor cells, PGHM-R-Fe could significantly upregulate the expression of ACSL4 and increase the production of ferroptosis-susceptible PUFAs. This increase in PUFAs further enhanced the sensitivity of tumor cells to ROS attack and improved the ferroptosis-dependent antitumor efficacy 18. Based on these studies, it is believed that increasing the enzymatic activity of ACSL4 will provide sufficient 'fuel' for LPO, which, in conjunction with the abundant ROS generated by SDT, could trigger effective ferroptosis tumor therapy. However, direct targeted delivery of composite nano formulations containing ACSL4 and sonosensitizers has not been reported, primarily due to the many unavoidable challenges related to their preparation process and stability.

Exosomes are extracellular nanovesicles (NVs) secreted by eukaryotic cells, ranging in size from 30-150 nm, and they play important roles in intercellular communication due to their nucleic acids and proteins content 19. Compared to traditional synthetic delivery vehicles, the surface-specific membrane proteins and lipids of tumor-derived exosomes promote their fusion with parental cells, facilitating efficient cellular targeting and uptake, and offering advantages such as low immunogenicity and favorable biocompatibility 20. Given these benefits, engineered exosomes derived from tumor cells can be developed as engineered exosomes to load a variety of therapeutic payloads, demonstrating excellent biosafety and therapeutic efficacy in applications such as CDT, PDT and SDT 21. However, previous studies have primarily focused on using tumor cell-derived exosomes as drug carriers to improve the tumor-targeting capabilities of therapeutics, while neglecting the inherent advantage of exosomes as endogenous carriers that can highly express multiple active molecules. Although some studies have attempted to load proteins into exosomes using passive loading methods such as electroporation, sonication and freeze-thaw cycling, these methods often result in low loading efficiencies, which significantly reducing protein bioactivity 22. Given the importance of proteins in regulating tumor microenvironment and activating ferroptosis, we hypothesized that engineered exosomes expressing endogenous functional proteins could serve as an ideal delivery system for SDT and ferroptosis therapies.

**Scheme 1:**
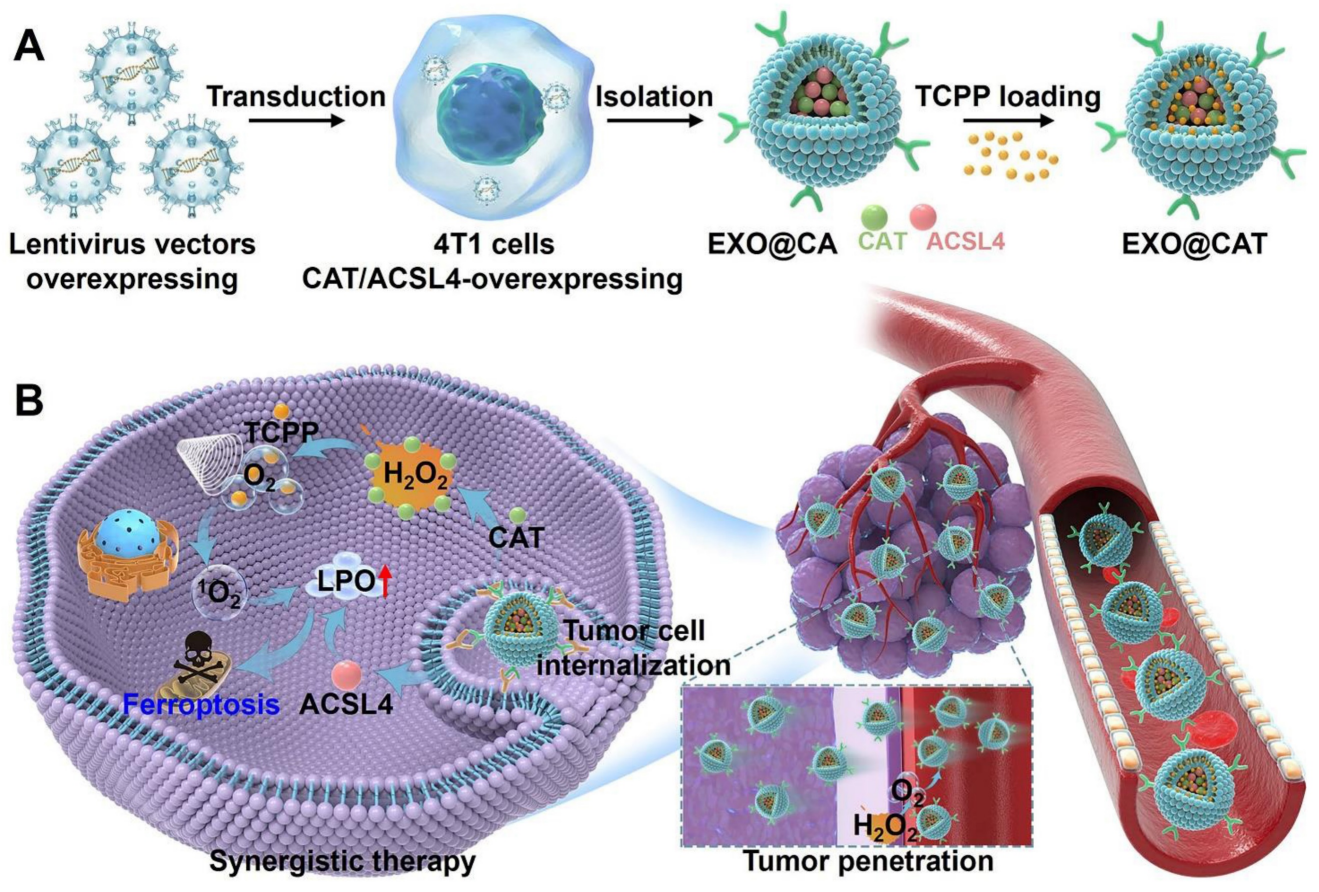
Schematic illustration of the preparation and treatment mechanisms of EXO@CAT NVs. (A) Preparation procedure of EXO@CAT NVs. (B) Active motion of EXO@CAT/US NVs improve blood vessel extravasation, tumor penetration and cell internalization, followed by synergistic treatment of tumour cells *via* SDT and ferroptosis.

In this study, we first constructed stable high expression of Cat and ACSL4 in 4T1 cells *via* lentivirus transfection. Subsequently, we extracted Cat and ACSL4 enriched exosomes (EXO@CA NVs) from the transfected cells, and further loaded the sonosensitizer tetrakis (4-carboxyphenyl) porphyrin (TCPP) through electroporation to obtain the engineered exosomes named EXO@CAT NVs (Scheme 1A). After intravenous injection, EXO@CAT NVs effectively accumulated in parental breast cancer tissues, where the Cat within the EXO@CAT NVs catalyzed the production of O_2_ from H_2_O_2_ in TME through the specific chemical catalysis between Cat and H_2_O_2_, thereby facilitating the active penetration of EXO@CAT NVs into the interior of the tumor. Leveraging the catalytic feature of Cat, EXO@CAT NVs could effectively alleviate tumor hypoxia after being internalized by tumor cells, and the therapeutic effect of SDT was further enhanced by the large amount of ^1^O_2_ produced by the sonosensitizer TCPP under US activation. Meanwhile, EXO@CAT NVs provided ACSL4, an important isozyme involved in the metabolism of PUFAs, contributing to an accumulation of LPO to render cancer cells more sensitive to ferroptosis (Scheme 1b). *In vivo* anti-tumor studies performed on 4T1 tumor-bearing mice demonstrated that the synergistic effect of O_2_-enriched SDT and ACSL4 effectively increased the sensitivity to ferroptosis, significantly inducing cancer cell death and inhibiting tumor proliferation. Therefore, this study not only provides an effective strategy to overcome the limitation of SDT application, but also provides a new insight into the efficient synergy between ferroptosis and SDT.

## Experimental methods

### Materials

TCPP, 3-(4,5-dimethylthiazol-2-yl)-2,5-diphenyltetrazolium bromide (MTT), trypsin, annexin V-FITC, Calcein-AM, propidium iodide (PI), 2′,7′-dichlorofluorescin diacetate (DCFH-DA), 1,3-diphenylisobenzofuran (DPBF), 4′,6-diamidino-2-phenylindole (DAPI), BioTracker FerroOrange, 3,3′-dioctadecyloxacarbocyanine perchlorate (DIO), and Cyanine 5.5 (Cy5.5) were offered by Sigma-Aldrich (Shanghai, China). Anti-Ki-67, anti-TUNEL, anti-Ferritin, anti-GPX4 and anti-SLC7A11 were provided by Abcam (Shanghai, China). Anti-CD63, anti-CD81, anti-TSG101, anti-ACSL4 and anti-Cat were obtained from Proteintech (Wuhan, China). BODIPY^581/591^-C11 was offered by Thermo Fisher (Waltham, MA). JC-1 Mitochondrial Assay Kit were obtained by Aladdin (Shanghai, China). Lentiviral vectors which overexpress Lentivirus encoding Cat (Lenti-Cat), ACSL4 (Lenti-ACSL4) and empty vector (Lenti-NC) were provided by Genechem Biosciences. Mouse Cat ELISA Kit was obtained from CUSABIO (Wuhan, China). Mouse ACSL4 ELISA Kit was purchased from Huabang BIO (Shanghai, China). All other chemicals and solvents at reagent grade or better were offered by Changzheng Regents Co. (Chengdu, China), unless otherwise explained.

### Construction and identification of Cat and ACSL4-overexpressing 4T1 Cells

4T1 cells were acquired from the American Type Culture Collection (Rockville, MD, USA) and cultured in 1640 media supplemented with 10% fetal bovine serum (Gibco BRL, USA) at 37 °C in a humidified atmosphere containing 5% CO_2_. The Cat and ACSL4 overexpressing 4T1 cells were generated by infecting 4T1 cells with Lenti-Cat and Lenti-ACSL4, as previously described 23. Selection of the infected cells was conducted using 3 × 10^-3^ mg/mL puromycin. Expression levels of Cat and ACSL4 in 4T1 cells were assessed by Western blot analysis. Briefly, the Cat and ACSL4-overexpressing 4T1 cells were lysed in RIPA buffer, and the total protein concentration was determined using a bicinchoninic acid (BCA) kit. Samples were resolved using 12% sodium dodecyl sulfate polyacrylamide gel electrophoresis (SDS-PAGE) and then transferred onto a nitrocellulose membrane. After blocking step with 5% nonfat milk for 1.5 h, the immunoblotting was performed at 4 °C for overnight using primary antibodies against Cat and ACSL4, and then incubation with horseradish peroxidase (HRP) secondary antibody for 1 h at room temperature. Protein signals were detected using a Gel Doc XR imaging system (Bio-Rad, Lab, Hercules, CA) and with GAPDH serving as a control.

### Preparation and characterization of EXO@CAT NVs

The EXO@CA NVs were purified from culture supernatant of Cat and ACSL4-overexpressing 4T1 cells using gradient ultracentrifugation as previously reported 24. Briefly, the 4T1 cells overexpressing Cat and ACSL4 were incubated in fetal bovine serum-free 1640 media for 24 h. The culture supernatant was centrifuged at 300 g and 2000 g for 10 min to remove cells and cell fragments, respectively. This was followed by a further centrifugation step at 10,000 g for 30 min to remove cell debris. Finally, the exosomes were obtained by centrifugation at 100000 g for 4 h at 4 °C. The obtained EXO@CA NVs were washed and resuspended in phosphate buffered saline (PBS). To obtain engineered EXO@CAT NVs, the TCPP molecules were encapsulated into EXO@CA *via* an electroporation procedure 25. Specifically, EXO@CA containing 100 μg of protein was diluted in PBS and mixed with 25 μg of TCPP in 800 μL of PBS. The mixture was incubated on ice for 10 min and then subjected to electroporation at 400 V using an electroporation instrument (Bio-Rad, USA). Afterwards, residual TCPP was removed by ultracentrifugation at 4000 g for 30 min using 100 kDa ultrafiltration centrifuge tubes (microporous, USA). Pure exosomes (EXO NVs) were extracted from Lenti-NC transfected 4T1 cells using the same protocol described above. The concentration of NVs was detected by the BCA method.

Morphological analysis of both EXO@CA NVs and EXO@CAT NVs was performed by transmission electron microscopy (TEM; FEI Tecnai F20, USA). The size distribution and zeta potential of NVs were measured using dynamic light scattering (DLS; Malvern Nano-ZS90, UK). The NVs were stored in different media for 14 days, and theie colloidal stability was determined by measuring hydrodynamic sizes and surface charges at different time points using DLS. The loading of TCPP was verified by confocal laser scanning microscopy (CLSM; Nikon A1R, Japan) following pre-labeling of EXO@CA with DIO. The loading rate and encapsulation efficiency of TCPP were calculated in comparison to serial TCPP solutions with known concentrations utilizing the ultraviolet-visible (UV-Vis) absorption spectrum (Shimadzu UV-2550, Japan) 26. The NVs lysate with the same protein concentration were analyzed *via* Western blotting. The primary antibodies used for this analysis included anti-CD63, anti-CD81, anti-TSG101, anti-ACSL4 and anti-Cat. The Cat catalytic activity of EXO@CAT, EXO@CA and free Cat were assessed after incubation with H_2_O_2_ solution for 10 min at pH 7.4 and 37 °C. One unit of Cat activity was defined as the amount of enzyme required to consume 1 μmol of H_2_O_2_ per minute, and the H_2_O_2_ concentration was measured by an UV-Vis spectrophotometer at 240 nm 27. *In vitro* release studies of TCPP, ACSL4, and Cat from the NVs were determined after incubation in buffers of pH 7.4 to simulate the pH values of blood. Briefly, NVs were placed in dialysis bags and incubated in release buffers at 37 °C. At predetermined time points, 1.0 mL of release buffer was retrieved to quantify the TCPP, ACSL4, and Cat levels, with an equal volume of fresh buffer added back for continuous incubation.

### Oxygen and ROS generation of EXO@CAT NVs

The oxygen generation capabilities of EXO@CAT NVs were examined using H_2_O_2_ as a substrate 28. In brief, suspensions of H_2_O_2_ (1.0 mM) and NVs (0.05 mg/mL) were incubated in PBS at 37 °C for 20 min. The oxygen generation was measured at different time points with a portable dissolved oxygen meter (Kolida Instrument, Guangzhou, China), employing PBS and pure H_2_O_2_ as the control. Subsequent to the incubation, the suspensions of NVs containing H_2_O_2_ were added to the aqueous 2,2,6,6-tetramethylpiperidine (TEMP) solution. The mixture was stimulated with or without US (1.0 MHz, 1.5 W/cm^2^) for 3 min, after which the production of ^1^O_2_ was monitored using an electron spin resonance (ESR) spectrometer (Bruker EMXplus) 29. Additionally, 1,3-diphenylisobenzofuran (DPBF) was used as a probe to detect the generation of ROS 30. Briefly, the suspensions of NVs were mixed with 0.5 mL of DPBF (1 mg/mL, ethanol solution) and maintained at 37 °C for 2 h in the dark. Subsequently, H_2_O_2_ (1.0 mM) was added, and the mixture was incubated for an additional 10 min before being subjected to US irradiation (1.0 MHz, 1.5 W/cm^2^) for 3 min. The absorbance at 410 nm was subsequently detected by UV-Vis spectroscopy.

### Motion profiles and penetration ability of EXO@CAT NVs

The motion profiles of EXO@CAT NVs were assessed in the presence of PBS containing H_2_O_2_ (1.0 mM), and compared with those of EXO@TCPP NVs and EXO@CA NVs. The motion trajectories of the NVs were recorded using confocal laser scanning microscopy (CLSM) at a frame rate of 25 fps. The coordinates of NVs was then tracked using ImageJ software, and analyzed through mean square displacement (MSD) analysis using the MATLAB software 31. To evaluate the penetration ability of the NVs, 3D tumor cell spheroids were constructed as 3D *in vitro* tumor models 32. Briefly, 3D tumor spheroids of 4T1 with diameters around 220-250 μm containing H_2_O_2_ (1.0 mM) were prepared in Corning's round bottom 96-wells plate with ultra-low attachment property. These spheroids were then incubated with Cy5.5-labelled NVs for 4 h. The Afterward, the tumor spheroids were fixed using 4% paraformaldehyde, and the images of different depths for tumor spheroids were obtained using Z-stack scanning at the excitation wavelength of 660 nm to clearness the permeation of NVs.

### Cellular uptake of EXO@CAT NVs

The cellular uptake assays were examined on 4T1 cells in the presence of H_2_O_2_ following treatment with EXO@CAT under US irradiation (EXO@CAT/US). Comparisons were made with EXO@TCPP/US, EXO@CA/US, EXO@CAT, and EXO as control groups. 4T1 cells (1 mL, 1 × 10^4^ cells per milliliter) were seeded into 24-well plates and incubated for 24 h. Following this, equivalent dosages of Cy5.5-labelled NVs containing H_2_O_2_ (1.0 mM) were added, and the incubation was continued for 4 h after 3 min of US irradiation. The collected cells were fixed with 4% paraformaldehyde for 15 min and subsequently stained with DAPI (excitation/emission wavelengths of 364/454 nm) for 10 min. Images were captured using confocal laser scanning microscopy (CLSM). Another batch of 4T1 cells was washed three times with PBS, and then flow cytometry analysis (Beckman CytoFLEX, USA) was used to quantify the cellular uptake after separation with trypsin 33.

### *In vitro* antitumor activity of EXO@CAT NVs

The *in vitro* antitumor activity was examined through cytotoxicity, intracellular ROS levels, live/dead staining, and apoptosis after treatment with EXO@CAT/US, using EXO@TCPP/US, EXO@CA/US, EXO@CAT, and EXO serving as controls 34. 4T1 cells containing H_2_O_2_ (1.0 mM) were cultured overnight in 96-well plates at a density of 6 × 10^3^ cells per well, and then treated with 100 μL of NVs at different concentrations from 0 to 0.15 mg/mL for 24 h. The parallel cell groups were irradiated with US (1.0 MHz, 1.5 W/cm^2^) for 3 min, and incubation for other 24 h. After that, MTT (20 μL, 5 mg/mL in PBS) was added into each well for a 4 h incubation. The medium was then replaced with 150 μL of DMSO, and the absorbance of the solution at 570 nm was measured to determine the *in vitro* viability of cells. Moreover, the viability of HepG2 (liver cancer), PC-3 (prostate cancer), and A549 (lung cancer) cells after treatment with EXO@CAT/US was also texted as above methods. Another batch of experiment was stained with Calcein-AM and PI for 20 min to distinguish viable and dead cells. After carefully washing with PBS, the cell images were observed by CLSM. For apoptosis analysis, treated 4T1 cells were collected by trypsin digestion and stained with annexin V-FITC (5 μL) and PI (10 μL) for 15 min before flow cytometry analysis. Moreover, flow cytometry was also utilized to detect intracellular ROS production according to the previously reported literature 35. The treated cells were resuspended with serum-free medium containing DCFH-DA (10 µM) for 0.5 h in the dark, and then the intracellular ROS production was detected by flow cytometry and CLSM analysis.

### Confirmation the ferroptosis of EXO@CAT NVs

BODIPY^581/591^-C11 as a fluorescent probe to confirm the intracellular LPO levels 36. 4T1 cells were treated with EXO@CAT/US, EXO@TCPP/US, and EXO@CAT NVs for 24 h, using PBS as a control. The cells were then incubated with 5 μM of BODIPY^581/591^-C11 for 20 min prior to detect by CLSM. Imaging was performed immediately, with green fluorescent oxidation products detected at excitation and emission wavelengths of 485 nm and 520 nm, respectively. The membrane potential of mitochondrial in the treated 4T1 cells was assessed using the JC-1 Mitochondrial Assay Kit. The excitation and emission wavelengths for green JC-1 monomers and red JC-1 aggregates were 488/510 nm and 561/590 nm, respectively. The treated 4T1 cells were further incubated with the fluorescent agent FerroOrange (3 μM) for 30 min to detect the cellular iron levels using CLSM, with excitation and emission wavelength set to 543 and 580 nm, respectively 37. After treatment of 4T1 cells by different methods, total proteins were extracted from lysates containing cocktail protease inhibitors, and Western blotting was performed to evaluate the expression levels of GPX4, Ferritin, and SLC7A11, with Tubulin as a control. In addition, before embedding in EPON 812 epoxy resin, samples were fixed in electron microscope fixative and the dehydration in graded ethanol at 70, 90, 96 and 100% before sectioning and positive staining with uranium and lead salts. The morphological variations of mitochondria were observed by bio-TEM (JEM-1400, JEOL, Japan) 38. A standard GSH assay was used to detect the changes in intracellular GSH levels after different treatments. Briefly, 4T1 cells were treated with EXO@CAT/US, EXO@TCPP/US, EXO@CAT NVs, and US for 3 min, and then incubation for 24 h. The cells were digested with trypsin and washed three times in PBS, and the test procedure was referred to the manufacturer's protocol of the GSH assay kit. The content of GSH was compared to that of untreated cells. Additionally, 4T1 cells at a density of 5 × 10^3^ cells per well in 96-well plates were cultured for 24 h, and subsequently treated with different NVs for another 24 h. The culture medium was then supplemented with ^1^O_2_ inhibitor (furfuryl alcohol: FFA, 100 μM), apoptosis inhibitor (Z-VAD-FMK, 30 μM), and ferroptosis inhibitor (Fer-1, 1 μM) for an additional 24 h incubation 39. Cytotoxicity before and after the addition of inhibitors was quantified by standard MTT assay.

### *In vivo* biodistribution study

The biodistribution of Cy5.5-labeled EXO@TCPP NVs and EXO@CAT NVs was determined in mouse models bearing breast tumor after intravenous injection 40. All animal experiments were performed following the Institutional Guide for the Care and Use of Laboratory Animals of China and the protocols were approved by the Animal Care and Use Committee of West China Hospital, Sichuan University (No: 20240223059). Briefly, 4T1 cells were inoculated into the mammary fat pad of female Balb/c mice obtained from Sichuan Dashuo Biotech. (Chengdu, China) to establish the tumor model. When tumors grew to approximately 100 mm^3^, mice were randomized into two groups and injected with NVs *via* tail veins (200 μg per mouse). The fluorescence images of mice were recorded by an IVIS imaging system (Caliper Life Sciences Inc., USA) after anesthesia. The major organs and tumors were collected to assess *ex vivo* biodistribution after 24 h administration. Additionally, photoacoustic imaging was detected to analyze oxygen distribution using a Vevo LAZR photoacoustic imaging system (Visualsonics, Toronto, Canada) equipped with a 21 MHz linear array transducer. The excitation wavelengths ranged from 680 and 970 nm at 100% power and 40 dB 41. DCFH-DA was used to detect intratumor ROS distribution according to the previous reports 42. Briefly, EXO@TCPP NVs or EXO@CAT NVs were injected intravenously into 4T1 tumor-bearing mice. 24 h later, DCFH-DA (100 μL, 50 μM) was orthotopically injected into tumors and irradiated with US (1.0 MHz, 1.5 W/cm^2^) for 3 min. Then, the treated tumors were harvested and sectioned for observation with CLSM. To examine the spatial distribution of NVs after 24 h administration, the tumors were frozenly cut into 5 μm-thick sections to stain with DAPI and anti-CD31 for 3 h, and incubate with IgG-FITC for 2 h. Fluorescence images were recorded using CLSM 43.

### *In vivo* antitumor evaluation of EXO@CAT NVs

Tumor-bearing mice were randomly divided into four groups (PBS, EXO@CAT, EXO@TCPP/US, and EXO@CAT/US; *n* = 4). Upon tumor volume reaching 100 mm^3^, the mice were intravenously injected with 100 μL of the appropriate solutions (2 mg/mL) 44. The mice receiving EXO@TCPP/US, and EXO@CAT/US underwent US treatment (1.0 MHz, 1.5 W/cm^2^) for 3 min after 24 h post-administration. The tumor size and body weight of mice were measured every two days, and the tumor volume was calculated by V = (L × W^2^)/2, where L and W represent the longest and shortest diameter of the tumor, respectively. After 14 days of treatment, the mice and the solid tumors were retrieved for taking photos. The tumors stained with hematoxylin and eosin (H&E) as staining protocol, and TUNEL and Ki-67 was performed to analyze the apoptosis and proliferation activity of tumors. Positively stained cells were counted under a light microscope and the total number of cells was normalized. In addition, the morphological variations of mitochondria of tumors were also tested through bio-TEM as previously described. After treatment with different methods, total proteins of tumors were extracted from lysates containing cocktail protease inhibitors, and Western blotting was performed to analyses the expression of GPX4, Ferritin, and SLC7A11, with Tubulin as a control.

### Biosafety of EXO@CAT

Biosafety was evaluated through hematological, biochemical analysis and histological examination of major organs 45. The blood was collected 24 h post-administration to analyze the blood markers (red blood cells, RBC; hemoglobin, HGB; white blood cells, WBC; platelets, PLT), and biochemical markers (blood urea nitrogen, BUN; aspartate aminotransferase, AST; L-lactate dehydrogenase, L-LDH; creatine kinase, CK) using Blood Biochemistry Analyzer (MNCHIP POINTCARE) and Auto Hematology Analyzer (MC-6200VET). The major organs (heart, lung, spleen, liver, and kidney) of the sacrificed mice were harvested and processed for H&E staining protocols for histological examination.

### Statistical analysis

The results are presented as mean ± standard deviation (SD). Where appropriate, multiple groups were compared by analysis of variance (ANOVA), and the statistical difference between the two groups was revealed by a two-tailed Student's t-test. A probability value (p) of less than 0.05 was considered statistically significant.

## Results and Discussion

### Characterization of EXO@CAT NVs

**Figure 1:**
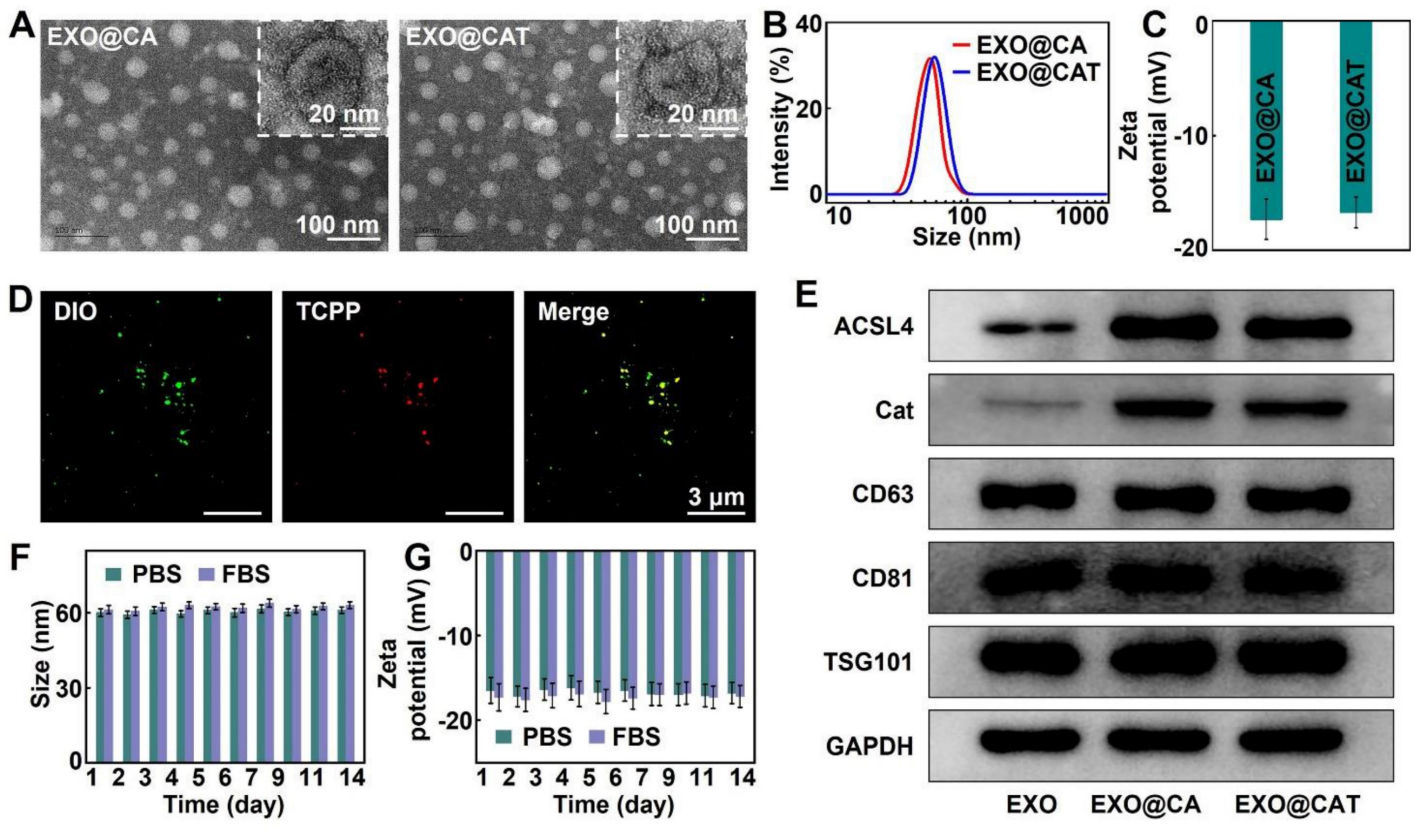
Characterization of EXO@CA and EXO@CAT NVs. (A) TEM images and inset pictures of high magnification TEM, (B) size distribution, and (C) zeta potential of EXO@CA and EXO@CAT NVs. (D) CLSM images of EXO@CAT NVs with DIO marking EXO@CA NVs. (E) Western blotting analysis of ACSL4, Cat, CD63, CD81, and TSG101 expression in EXO, EXO@CA and EXO@CAT NVs. (F) hydrodynamic size and (G) zeta potential changes of EXO@CAT NVs after incubation for 14 days in PBS and FBS (*n* = 3).

To verify the effective transfection of lentivirus vectors, we analyzed the differences in the expression of Cat and ACSL4 in cells by Western blot analysis. Figure S1A and S1B showed that Cat and ACSL4 were highly expressed in Lenti-Cat and Lenti-ACSL4 transfected cells compared to Lenti-NC transfected cells (p < 0.05), indicating the successful engineering of 4T1 cells. The EXO@CA NVs were obtained by extracting exosomes from 4T1 cells with high expression of Cat and ACSL4, and the engineered exosomes EXO@CAT NVs were further obtained by loading TCPP into EXO@CA NVs using electroporation. TEM images revealed that EXO@CA NVs and EXO@CAT NVs were almost typical spherical vesicle structures and uniformly distributed, with diameters of less than 45 nm, indicating that the electroporation process did not disrupt the structural integrity of EXO@CA NVs (Figure 1A). DLS measurements showed a slight increase in the average hydrodynamic diameter of the EXO@CAT NVs after TCPP loading, measuring 59 ± 5 nm, compared to 52 ± 3 nm for EXO@CA NVs (Figure 1B). Additionally, the zeta potentials of both EXO@CA NVs and EXO@CAT NVs were almost identical, approximately -17.0 ± 2.1 mV (Figure 1C). As shown in Figure 1D, the DIO fluorescence labeling of NVs appeared bright green, while TCPP exhibited bright red fluorescence. The presence of an obvious orange in EXO@CAT NVs, resulting from the combination of green and red fluorescence, indicated the successful loading of TCPP into the NVs. The encapsulation efficiency was calculated as 47.79 ± 3.25% with the drug-loading ratio of 6.85 ± 0.52% for EXO@CAT NVs. The quantification of TCPP loading in EXO@CAT was 16 μg/mg protein through UV-Vis absorption spectrum, while Cat and ACSL4 were 131 pg/mg protein and 246 pg/mg protein through ELISA method, respectively, and the Cat and ACSL4 in EXO were 18 pg/mg protein and 31 pg/mg protein. To identify the protein expression of exosomes, Western blotting was used to verify the presence of typical exosome protein markers and functional proteins. As shown in Figure 1E and S2A, exosome-specific markers CD63, CD81 and TSG101 were clearly detected in all samples, which confirms the successful isolation of exosomes 46. More importantly, the expression of ACSL4 and Cat in EXO@CA NVs and EXO@CAT NVs was positive compared to pure EXO NVs, confirming the presence of functional proteins in EXO@CAT NVs and EXO@CA NVs. In addition, the catalytic activity of EXO@CA and EXO@CAT still maintained over 90%, compared to that of the free enzyme (Figure S2B). Moreover, as shown in Figure S2C, less than 10% of TCPP, ACLS4, and Cat was released after 24 h of incubation in pH 7.4, suggesting that NVs could prevent drug release during blood circulation and thereby increase drug availability to tumors. The biostability of drug carriers is a key factor for maintaining their structural integrity during *in vivo* circulation 47. To evaluate this, we investigated the long-term storage stability and serum stability of EXO@CAT NVs by monitoring changes in particle size and zeta potential. As shown in Figure 1F and G, no significant change was observed in the particle size and zeta potential of EXO@CAT NVs after 14 days of storage in PBS and PBS containing 50% fetal bovine serum (FBS) (p > 0.05). These results indicated that EXO@CAT NVs possesses good stability, which is conducive to its *in vivo* application.

### Oxygen and ROS generation of EXO@CAT NVs

**Figure 2:**
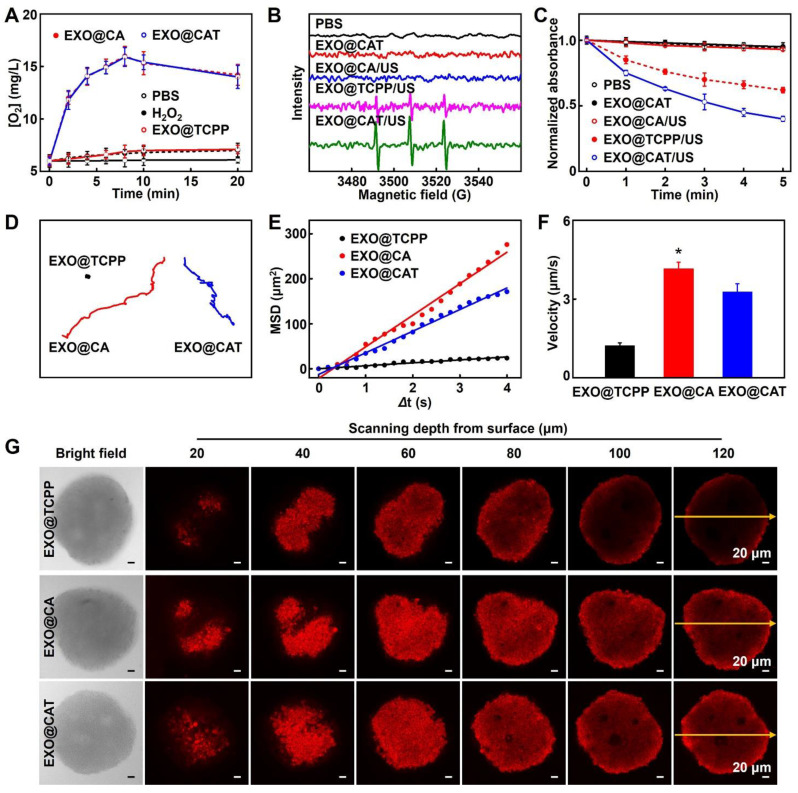
Oxygen and ROS generation, and the motion profiles of EXO@CAT NVs with H_2_O_2_. (A) Oxygen levels after incubation with different NVs (*n* = 3). (B) ESR spectra for ^1^O_2_ examination after treatment with NVs. (C) Absorbance changes of DPBF solutions after incubation with NVs. (D) Tracking trajectories, (E) average MSD values versus time intervals (*Δ*t), (F) motion velocities (*n* = 20, *: p < 0.05 *vs* other treatments), and (G) *in vitro* penetration results in 3D tumor spheroids of EXO@TCPP, EXO@CA and EXO@CAT NVs.

The oxygen levels produced by EXO@CAT NVs in the presence of H_2_O_2_ were summarized in Figure 2A. EXO@TCPP NVs mixed with H_2_O_2_ showed negligible oxygen production within 20 min, comparable to pure H_2_O_2_ (approximately 7.0 × 10^-3^ mg/mL). As expected, the EXO@CA NVs and EXO@CAT NVs containing Cat caused a rapid increase in oxygen levels, reaching 15.3 × 10^-3^ mg/mL, and then the oxygen levels subsequently decreased slowly due to the depletion of H_2_O_2_. These results indicated that the Cat in EXO@CA NVs and EXO@CAT NVs can effectively catalyze the production of oxygen from H_2_O_2_, which implies that engineered exosomes can be used to alleviate hypoxia by catalyzing H_2_O_2_ decomposition in the tumor microenvironment, potentially enhancing the efficacy of SDT. Subsequently, ESR spectroscopy was used to verify the production of ^1^O_2_ with TEMP as the trapping agent 29, while the peaks of TEMP-^1^O_2_ with intensities in a 1:1:1 ratio indicating the production of ^1^O_2_. After co-incubation of EXO@CAT NVs or EXO@CA NVs with H_2_O_2_ under US irradiation, an apparent three-line ESR signal of TEMP-^1^O_2_ adducts was observed, whereas little change in TEMP-^1^O_2_ intensity was observed for EXO@CA/US, EXO@CAT NVs and PBS with H_2_O_2_ (Figure 2B). Notably, the TEMP-^1^O_2_ intensity for EXO@CAT/US was significantly higher than that of EXO@TCPP/US, due to the greater amount of O_2_ produced by EXO@CAT NVs. In addition, the ^1^O_2_ generation capacity of EXO@CAT NVs under US irradiation was also investigated using UV-Vis spectroscopy with DPBF as the indicator. As shown in Figure 2C, a decrease in DPBF absorption was observed in EXO@CAT/US and EXO@TCPP/US with H_2_O_2_, while EXO@CA/US, EXO@CAT NVs, and PBS showed no changes in DPBF absorption, indicating the ultrasonication-dependent generation of ^1^O_2_ by TCPP. Above results indicating that TCPP loaded on EXO@CAT NVs could generate ^1^O_2_ under US irradiation, and the oxygen generation facilitating the production of ^1^O_2_.

### Motion profiles of EXO@CAT NVs

Considering that the oxygen produced during co-incubation of EXO@CAT NVs with H_2_O_2_ is the driving force for exosome movement, we next investigated the movement performance of exosomes *in vitro*, including tracking movement trajectories, calculating MSD values, and measuring velocities, which are essential for predicting the possibility of their future movement *in vivo*. Figures 2D and S3A showed the trajectories of exosomes in PBS containing H_2_O_2_, and it can be clearly observed that EXO@CAT NVs and EXO@CA NVs produced oxygen, which resulted in a pronounced motion behavior, whereas EXO@TCPP NVs displayed typical Brownian motion due to the lack of driving force. The EXO@CAT NVs exhibited the lower MSD values (Figure 2E) and velocity (Figure 2F) than that of EXO@CA NVs, likely because the inclusion of TCPP in EXO@CAT NVs increased the total weight. The *in vitro* movement results suggested that the motility of engineered exosomes may facilitate their intercellular transport. Therefore, we further constructed 3D tumor spheroids as *in vitro* tumor models to study the permeability of NVs for 4 h. Compared with the penetration distance of EXO@TCPP NVs, the EXO@CAT NVs and EXO@CA NVs display much deep penetration depth (Figure 2G). As shown in S3B, the fluorescence intensity of EXO@CAT NVs was about 37 a.u. across the 120 µm section, while the fluorescence intensity of EXO@TCPP NVs was only 11 a.u. These results indicated that the oxygen generation could enhances the motion performance of exosomes, thereby facilitating their penetration into 3D tumor spheroids.

### Cellular uptake of EXO@CAT NVs

Cellular uptake of exosomes with H_2_O_2_ adding by 4T1 cells was detected using CLSM and flow cytometry. The CLSM images of Cy5.5-labelled NVs after co-incubation with DAPI-stained 4T1 cells were shown in Figure S4A. EXO@CAT/US exhibited brighter red signals compared to EXO@TCPP/US, likely due to increased motion that enhanced the opportunities for contact with 4T1 cells. The fluorescence signals showed no difference between EXO@CAT/US and EXO@CA/US. In addition, the red signals for EXO@CAT/US were also stronger than that for EXO@CAT NVs treatment, indicating that the ultrasonication treatment could be beneficial for cell phagocytosis. Moreover, the EXO@CAT/US showed significantly stronger red signals than EXO NVs treatment due to the combined effect of motion and ultrasonication. The flow cytometry analysis for 4T1 cells was shown in Figure S4B. The fluorescence intensity of cells treated with EXO@CAT/US showed no significant difference when compared to those treated with EXO@CA/US. However, the fluorescence intensity for EXO@CAT/US treatment was 1.7- and 2.1-fold higher than that for EXO@TCPP/US and EXO@CAT NVs (p < 0.05), respectively. These results clearly indicated that both the ultrasonication and motion of EXO@CAT NVs promoted cell internalization.

### *In vitro* antitumor activity of EXO@CAT NVs

**Figure 3:**
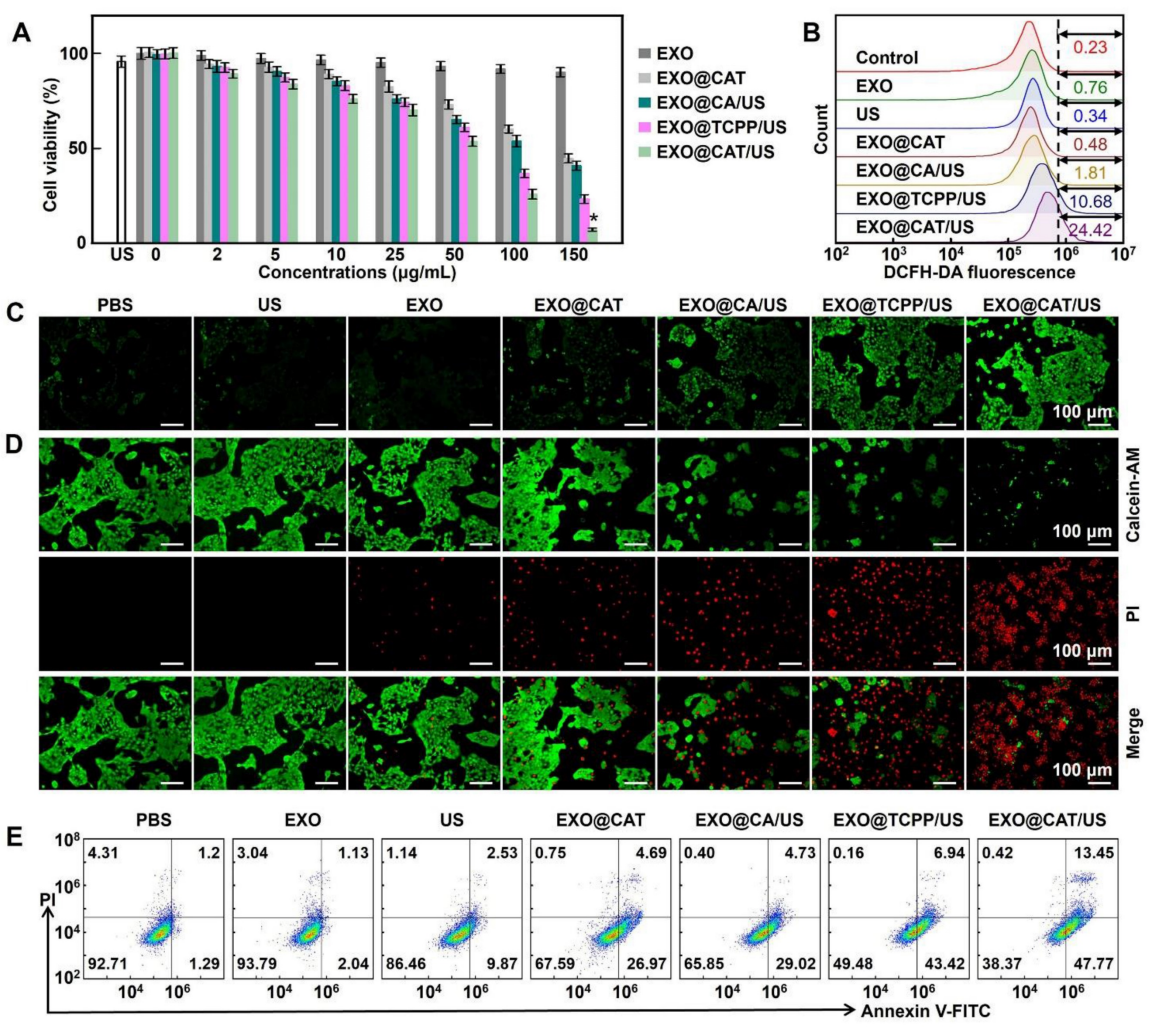
*In vitro* antitumor activity of EXO@CAT NVs. (A) 4T1 cell viability, (B) flow cytometry analysis of DCFH-DA-stained 4T1 cells, (C) CLSM images of DCFH-DA-stained 4T1 cells, (D) CLSM images of calcein-AM and PI-stained 4T1 cells, and (E) apoptosis assay of 4T1 cells after treatment with different NVs (*n* = 5, *: p < 0.05 *vs* other treatments).

The toxicity of EXO@CAT/US was evaluated in various cancer cell lines, including 4T1, HepG2, PC-3, and A549 cells. As shown in Figure 3A, the treatment with EXO@CAT, EXO@CA/US, EXO@TCPP/US, and EXO@CAT/US showed a dose-dependent cytotoxicity, and the cell viabilities at a high concentration of 0.15 mg/mL decreased to 44.7%, 40.9%, 23.3% and 7.2%, respectively, while the treatment with US and EXO alone displayed negligible cytotoxicity. The combination of SDT and ferroptosis showed significantly lower cell viabilities after treatment with EXO@CAT/US compared to EXO@TCPP/US, which involved only SDT treatment. Both EXO@CAT and EXO@CA/US treatments resulted in similar cell viabilities when ferroptosis was the only modality employed. Figure S5A showed a significant difference in the half maximal inhibitory concentration (IC50) of different treatments. Under US-on conditions, the IC50 of EXO@CAT, EXO@TCPP, and EXO@CA was 35.8, 58.9, and 111.4 μg, respectively, which was around 4.3, 2.6 and 1.4-fold lower than that of EXO@CAT without US (153.7 μg). In addition, the cell viabilities of HepG2, PC-3, and A549 cells also showed a dose-dependent cytotoxicity after treatment with EXO@CAT/US (Figure S5B). Figure 3B showed the fluorescence intensity of probe for detection the intracellular ^1^O_2_ production. The EXO, EXO@CAT, and EXO@CA/US showed similar fluorescence intensity with the control and US groups, indicating no significant generation of ^1^O_2_. In contrast, the EXO@TCPP/US showed a significant enhancement in fluorescence intensity, while EXO@CAT/US showed 2.3-fold higher than that of EXO@TCPP/US, indicating that TCPP could produce ^1^O_2_ under ultrasonication and the oxygen generation could improve the efficiency of ^1^O_2_ generation. Moreover, the fluorescence intensity of CLSM images of DCFH-DA-stained 4T1 cells showed the similar results as above (Figure 3C). The live/dead cells staining results indicated that the cells treated with EXO and US remained alive, while 4T1 cells could be destructed by ferroptosis and SDT after EXO@CAT, EXO@CA/US and EXO@TCPP/US treatment (Figure 3D). The strongest red fluorescence and almost no viable cells were observed following treatment with EXO@CAT/US, attributed to the synergistic effects of SDT and ferroptosis. The flow cytometry analysis of apoptotic cells after cells were stained with annexin V-FITC and PI (Figure 3E). Most of the cells treated with US and EXO NVs remained alive, exhibiting apoptosis rates similar to that of the PBS treatment (lower than 4%). In contrast, the treatment with US, EXO@CAT, EXO@CA/US, EXO@TCPP/US, and EXO@CAT/US significantly increased apoptosis rates to 12.4%, 31.7%, 33.7%, 50.4%, and 61.2%, respectively, demonstrating the synergistic efficacy of SDT and ferroptosis of EXO@CAT/US NVs.

### Confirmation the ferroptosis of EXO@CAT NVs

**Figure 4:**
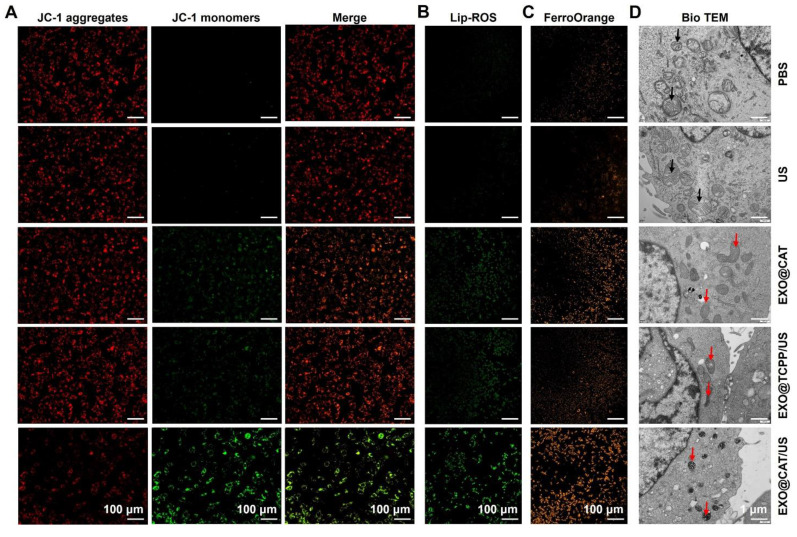
Confirmation of the ferroptosis of EXO@CAT NVs in 4T1 cells. (A) CLSM images of mitochondria membrane potential analysis by JC-1 staining, (B) CLSM images of cellular LPO by BODIPY^581/591^-C11 staining, (C) CLSM images of cellular Fe^2+^ by FerroOrange, and (D) bio-TEM images of 4T1 cells after different treatment, black arrow: normal mitochondria, red arrow: damaged mitochondria.

Membrane-permeable JC-1 dye serves as a probe for detecting the abnormal alterations in membrane potential, while the red fluorescent JC-1 aggregates accumulated in the healthy mitochondrial matrix and green JC-1 monomers indicated the occurrence of mitochondrial dysfunction 48. As shown in Figure 4A, the red fluorescence was decreased after treatment with EXO@CAT/US, EXO@TCPP/US, and EXO@CAT, compared to the PBS control and US group with predominantly red fluorescence, while the green fluorescence in cells was increase after the same treatments, indicating that the SDT and ACSL4 reduced the mitochondrial depolarization and maintained the high membrane potential of mitochondria. The ratios of red fluorescent JC-1 aggregates to green fluorescent JC-1 monomers were semi-quantitatively analyzed and showed in Figure S6A, EXO@CAT/US treatment was significantly lower than other groups. The LPO in cells are key determinants of cell lethality and important biomarkers of ferroptosis 49. BODIPY^581/591^-C11 was used as a fluorescent probe to evaluate the intracellular LPO levels 36. Figures 4B and S6B illustrated that the treatment with EXO@CAT/US resulted in distinct green fluorescence on the cell membrane when compared to EXO@TCPP/US, EXO@CAT, US, and PBS treatments, indicating the elevated production of LPO in the cells. These results were consistent with the results obtained by JC-1 dye. After EXO@CAT/US treatment, the FerroOrange signal increased significantly in 4T1 cells, indicating increased level of Fe^2+^ in the cytoplasm, which can induce ferroptosis 37. In contrast, treatments with EXO@TCPP/US and EXO@CAT caused few accumulations of Fe^2+^ in the cytoplasm (Figures 4C and S6C). In addition, the significant down-regulation of GPX4, Ferritin and SLC7A11 expression after EXO@CAT/US treatment is also strong evidence for the activation of ferroptosis (Figure S6D and E) 50. As can be seen from Figure S6F, 4T1 cells treated with EXO@CAT (69.5%), EXO@TCPP (60.3%), and EXO@CAT/US (28.3%) showed relatively lower intracellular GSH levels compared to the US- and PBS-treated control group. It is well known that ferroptosis involves the accumulation of LPO in mitochondria, resulting in severe damage to mitochondrial morphology 51, which could be further examined using bio-TEM. As shown in Figure 4D, the treatments with EXO@TCPP/US, EXO@CAT, and EXO@CAT/US showed typical features of ferroptosis, including shrunken mitochondria and reduced mitochondrial cristae, while control and US cells without any treatment retained normal cell morphology and their linear or granular mitochondria maintain intact bilayer membrane structure 52. These data confirmed that EXO@CAT could effectively trigger ferroptosis through a synergistic mechanism involving SDT-induced ROS production and ACSL4-mediated LPO.

To clarify the death mode induced by EXO@CAT/US, 4T1 cells were treated with FFA (^1^O_2_ inhibitor), Z-VAD-FMK (apoptosis inhibitor), and Fer-1 (ferroptosis inhibitor), respectively, prior to the cytotoxicity assay. As shown in Figure S6G, the cytotoxicity of cells treated with EXO@CAT/US in the presence of FFA, Z-VAD-FMK, and Fer-1 was alleviated to varying degrees, and the Fer-1-induced rescue was more pronounced than that induced by the addition of FFA and Z-VAD-FMK. In addition, the rescue effects of FFA and Z-VAD-FMK were comparable, indicating that the production of ^1^O_2_ may induce the cell death by apoptosis. These results suggested that the EXO@CAT/US-induced cell death pathway may involve both apoptosis and ferroptosis, and that the ferroptosis dominates relative to apoptosis.

### *In vivo* biodistribution study

**Figure 5:**
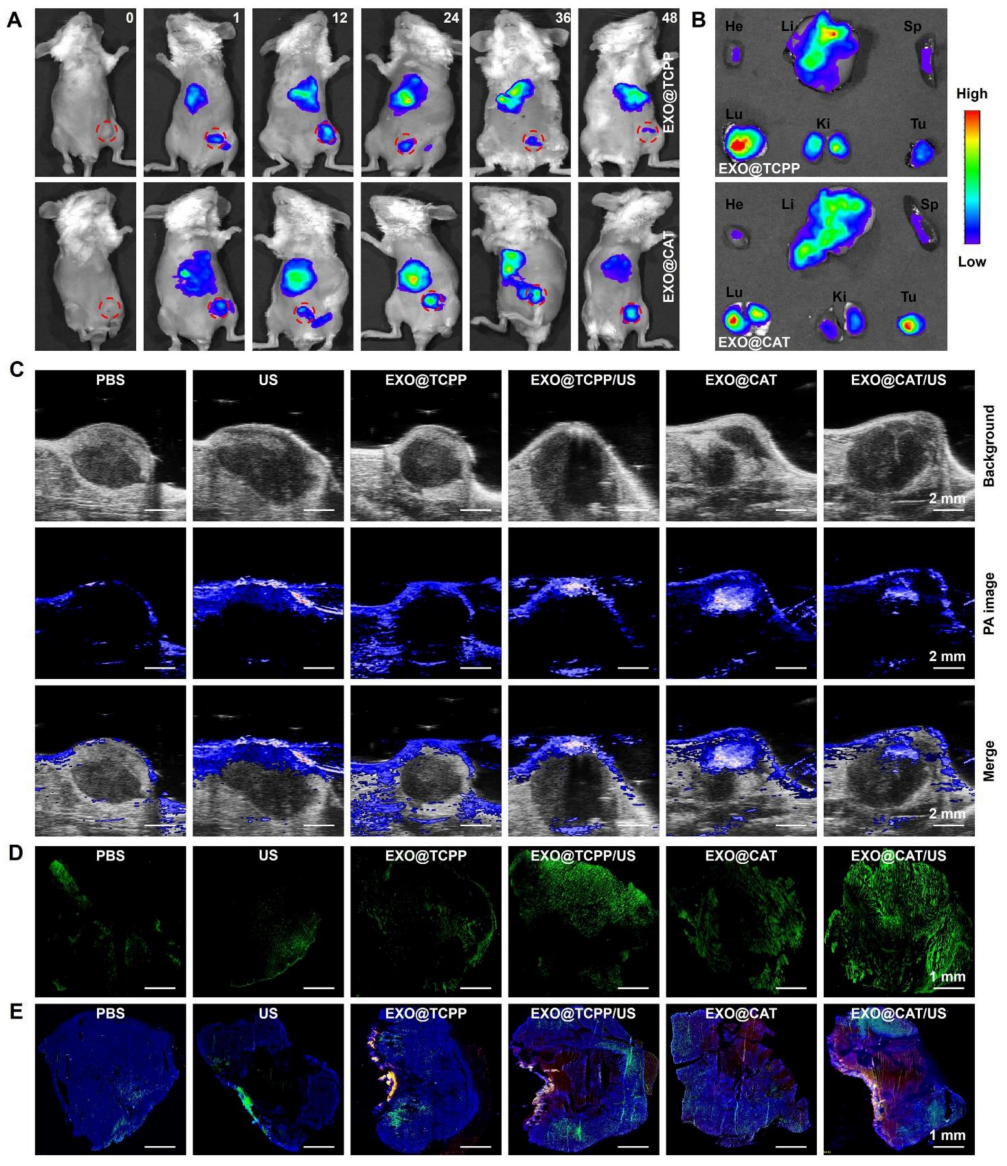
*In vivo* biodistribution study of NVs. (A) *In vivo* fluorescence images of tumor-bearing mice for various time intervals (red circle represents the location of tumor) and (B) *ex vivo* fluorescence images of major organs and tumor after intravenous injection with Cy5.5-labeled NVs for 24 h. He: heart; Li: liver; Sp: spleen; Lu: lung; Ki: kidney; Tu: tumor. (C) photoacoustic oxygen images of tumors, (D) stitched CLSM images of the whole tumor sections stained with DCFH-DA, and (E) stitched CLSM images of NP distributions (red) in tumors counterstained with DAPI (blue) and immunohistological staining on CD31 (green) after 24 h of different treatments.

The biodistribution of Cy5.5-labeled NVs was investigated using the IVIS imaging system to verify the precise tumor targeting performance, which is a key factor to enhance the antitumor effect of NVs. As illustrated in Figure 5A and S7, the fluorescent signal of Cy5.5 was rapidly and widely distributed *in vivo* after intravenous injection of EXO@TCPP and EXO@CAT NVs. EXO@TCPP and EXO@CAT showed significantly enhanced fluorescence signal in the tumor and peritumoral after 24 h post-administration, suggesting that the engineered exosomes could circulate and maintain high stability *in vivo*, allowing for precisely accumulate at the tumor site. The fluorescence signal was decreased from 24 to 36 h, and further reduction after 48 h. In addition, the fluorescence signal of EXO@CAT in tumor was stronger than EXO@TCPP after 36 and 48 h post-administration, suggesting that the self-propelled motion of EXO@CAT NVs facilitated tumor accumulation. Furthermore, *ex vivo* fluorescence imaging was obtained to further assess the biodistribution of tumors and major organs after 24 h post-injection (Figure 5B). Notably, the NVs were also distributed in liver, spleen, and kidney, which may be attributed to their involvement in metabolism and elimination 53, although the fluorescence intensity in these organs was relatively weak. These results strongly indicated that efficient accumulation of NVs at the tumor site will further inhibit tumor progression.

Photoacoustic imaging of oxygen levels was shown in Figure 5C. The photoacoustic signals of oxygen at the tumor area were significantly enhanced after injection of EXO@CAT NVs, demonstrating a uniform distribution of oxygen in tumor site. This finding suggested the effective accumulation and penetration of EXO@CAT NVs at the tumor site driven by the targeting and motion profiles. However, after 3 min of ultrasonication, the photoacoustic signals of EXO@CAT diminished, likely due to the consumption of oxygen by the SDT. In addition, the EXO@TCPP treatment showed no significant differences in the solid tumors compared to PBS control and US group. EXO@TCPP/US showed a slight enhancement of photoacoustic signals for that ultrasonication may increase the supplying of oxygen 54. These results suggest that EXO@CAT could significantly alleviate the hypoxic conditions at the tumor site.

To evaluate the generation of ROS, DCFH-DA was locally injected in solid tumors and assessed by CLSM. As shown in Figure 5D, the intensity of green fluorescence in EXO@CAT group was significantly higher than that in the EXO@TCPP, US and PBS group, which perhaps due to EXO@CAT provided more oxygen. However, strong green fluorescence was observed after ultrasonication treatment for EXO@TCPP/US and EXO@CAT/US, suggesting the SDT could increase the generation of ROS. In addition, EXO@CAT NVs could consistently produce oxygen and abundant accumulate in tumors. The self-propelled motion profiles of EXO@CAT NVs could enlarge spatial distributions of the generated oxygen, resulting in enhanced fluorescence intensities and fluorescence distribution of ROS in tumors compared to EXO@TCPP after ultrasonication.

Figure 5E displays the CLSM images to show the blood vessels with green signals and the spatial distribution of NVs with red signals, using DAPI staining to label the tumor sections. The strong red fluorescence was observed in the tumor after treatment with NVs, indicative of their precise tumor targeting performance. Moreover, the EXO@CAT NVs treatment showed stronger and better red fluorescence distribution, along with superior penetration ability of NVs across blood vessels than that of EXO@TCPP NVs. US activation of EXO@CAT NVs and EXO@TCPP NVs exhibited improved results than EXO@CAT and EXO@TCPP, respectively, indicating that the oxygen-propelled motion efficiently facilitated tumor accumulation, distribution and diffusion of NVs away from blood vessels. Thus, using EXO@CAT/US as a mobile platform could alter the vascular permeability and tissue diffusivity, leading to high tumor accumulation and distribution of NVs at tumor sites.

### *In vivo* antitumor evaluation of EXO@CAT NVs

**Figure 6:**
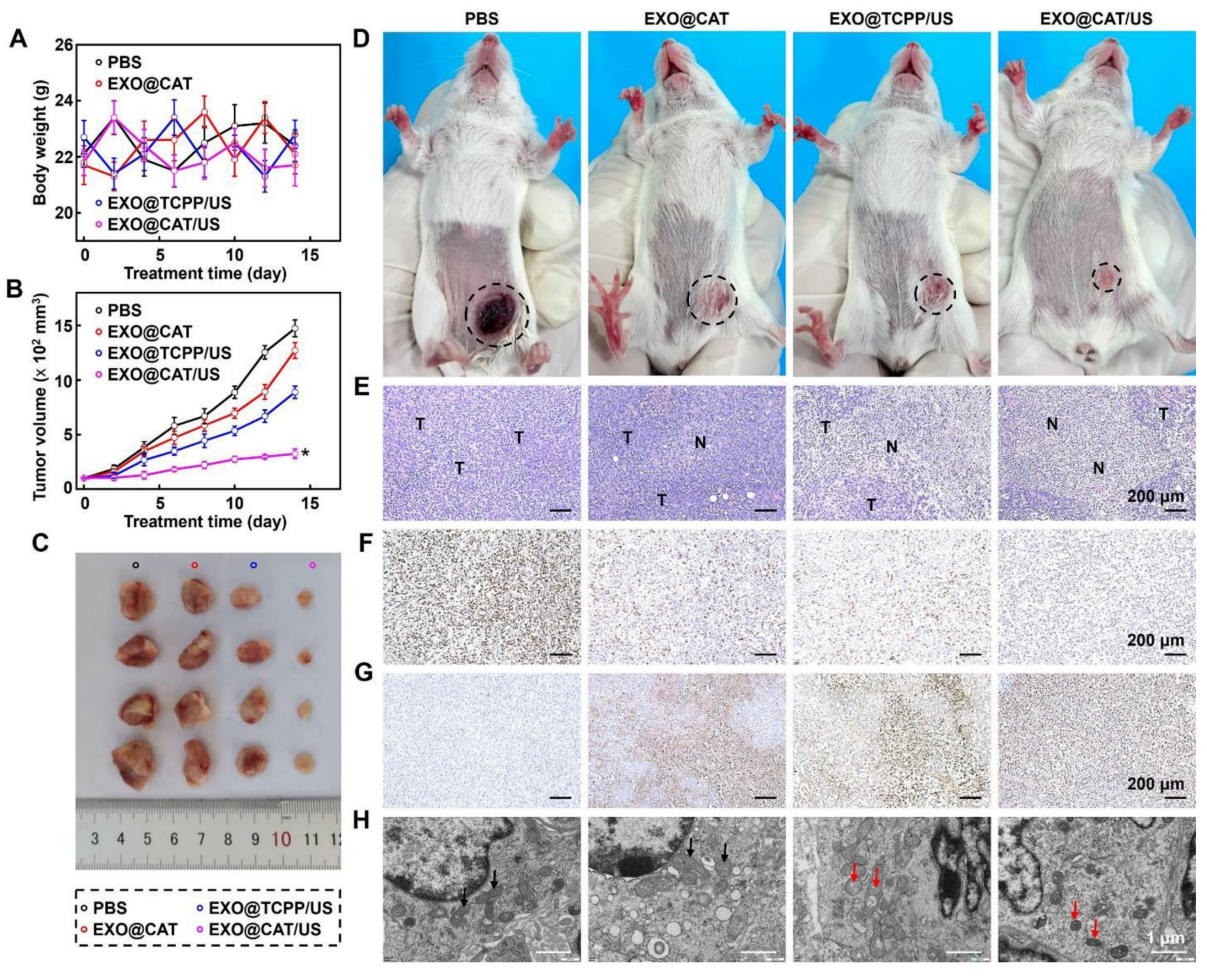
Antitumor efficacy of EXO@CAT NVs. (A) Body weight changes of 4T1 tumor-bearing mice, (B) tumor growth curves, (C) *in vitro* representative pictures of tumors, (D) *in vivo* representative pictures of 4T1 tumor-bearing mice (black circle represents the size and location of tumor), (E) H&E staining images (“N” represents necrotic area, “T” represents tumor mass), (F) Ki-67 staining images, (G) TUNEL staining images, and (H) bio-TEM in tumor sections after different treatments for 14 days, black arrow: normal mitochondria, red arrow: damaged mitochondria (*n* = 4, *: p < 0.05 *vs* other treatments).

Based on the excellent tumor accumulation, distribution and diffusion of EXO@CAT, we further investigated the tumor growth, histological and immunohistochemical (IHC) staining to assess the anticancer efficacy of NVs. As shown in Figure 6A, the body weights showed no significant changes in all the group during the whole experimental period, indicating the excellent biocompatibility of the NVs. The results for the tumor volume confirmed that treatments with ferroptosis of EXO@CAT NVs and SDT of EXO@TCPP/US slight inhibited the tumor volume to 1270 mm^3^ and 888 mm^3^, respectively, compared to a rapid growth to 1472 mm³ observed in the PBS control group (Figure 6B). In contrast, the synergy therapy of SDT and ferroptosis using EXO@CAT/US apparently inhibited tumor expansion to 324 mm^3^ after 14 days, showing the best tumor inhibition both in terms of tumor size and representative pictures *in vitro* (Figure 6C) and *in vivo* (Figure 6D).

To further investigate the microcosmic therapeutic efficacy, H&E, Ki-67, TUNEL staining, and bio-TEM of tumor sections were measured after different treatments with 14 days. EXO@CAT/US treatment showed obvious vacuolization and nucleus shrinkage, as well as more significant tumor cell destruction and larger necrotic areas compared with other treatments (Figure 6E). In addition, a similar trend was observed in Ki-67 and TUNEL staining images, which assessed the proliferative and apoptotic cells, respectively. As shown in Figure 6F, the cells proliferative rate of EXO@CAT/US treatment was at 7.9%, which was significantly lower than those observed in the EXO@TCPP/US (48.7%), EXO@CAT (59.9%), and PBS (90.3%) groups (p < 0.05). Furthermore, a higher percentage of apoptotic cells (84.6%) were counted after EXO@CAT/US treatment compared with the EXO@TCPP/US (61.3%), EXO@CAT (49.7%), and PBS (9.6%) groups (Figure 6G). The bio-TEM imaging revealed the mitochondrial damage characteristic of ferroptosis, including mitochondrial shrinkage and volume reduction 52. As shown in Figure 6H, tumor cells treated with EXO@CAT/US showed dual morphological features of ferroptosis and apoptosis with smaller mitochondria and distinct chromatin condensation, indicating that the EXO@CAT/US treatment could induced ferroptosis by increased LPO. In addition, the significant down-regulation of GPX4, Ferritin and SLC7A11 expression after EXO@CAT/US treatment is also strong evidence for the activation of ferroptosis (Figure S8A and B). All these results clearly confirmed that the superior tumor inhibition efficacy of EXO@CAT/US combining SDT and ferroptosis.

### Biosafety of EXO@CAT

**Figure 7:**
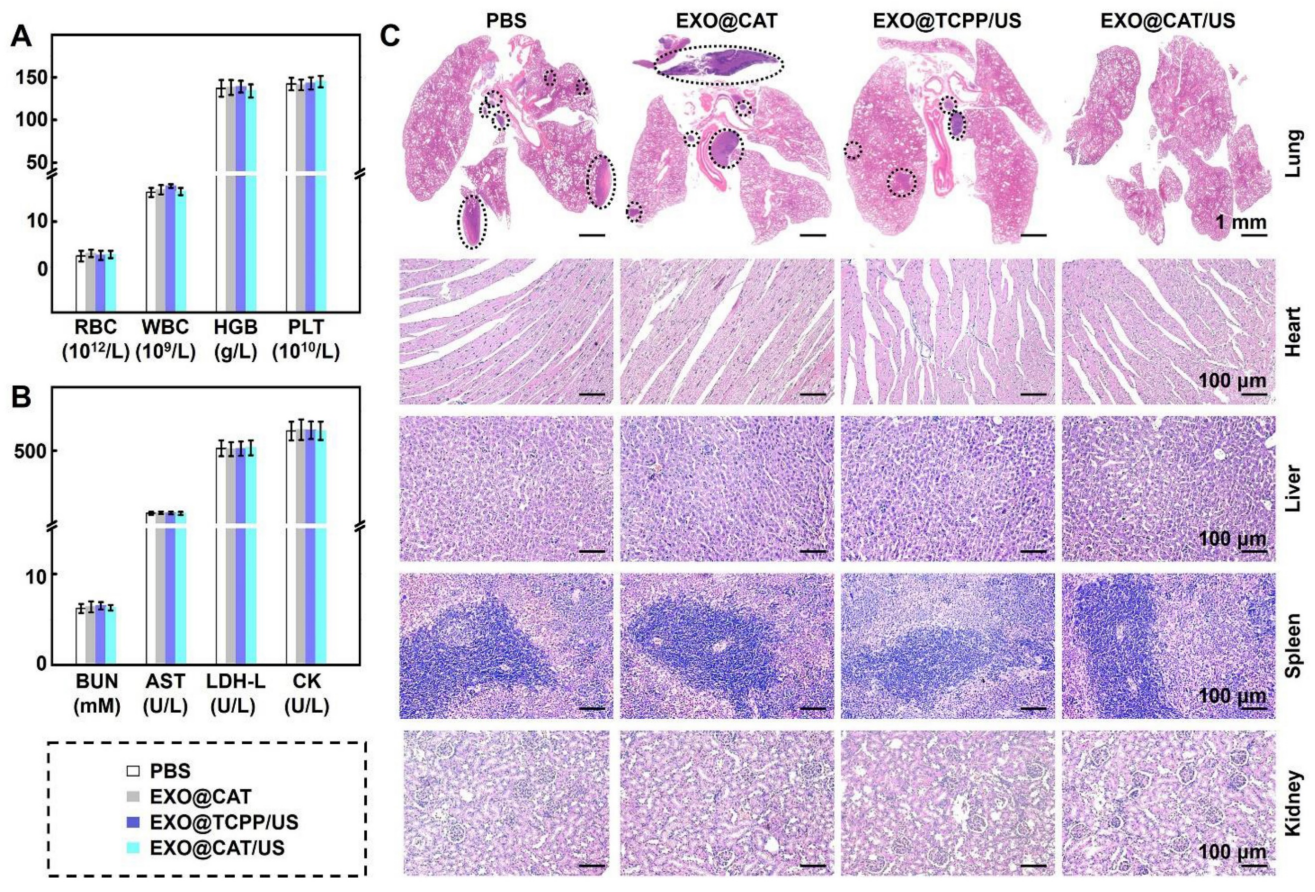
Treatment safety of EXO@CAT NVs. (A) Hematological (RBC, WBC, HGB, and PLT counts), (B) biochemical analyses (AST, BUN, L-LDH, and CK levels), and (C) H&E staining images of the heart, liver, spleen, lung and kidney after treatment with different NVs for 14 days (black circle represents the tumor location of lung metastasis; *n* = 3).

The biosafety of the treatment was examined through blood analysis and H&E staining of the major tissues after EXO@CAT/US treatment. Hematological examination results indicated that there were no significant changes compared with those of control groups after EXO@CAT/US treatment in the levels of RBC, WBC, HGB, and PLT (Figure 7A). In addition, serum biochemical parameters such as BUN, AST, L-LDH, and CK, which reflect the liver and kidney function, remained within the normal reference range (Figure 7B). The H&E staining images of major organs (liver, heart, kidney, and spleen) also demonstrated that there were no obvious structural disorders, degeneration, necrosis, and inflammatory infiltration compared to the PBS control group, indicating the favorable biocompatibility of EXO@CAT NVs. Notably, various numbers of metastatic tumors were found in the lungs of the PBS, EXO@CAT and EXO@TCPP/US groups, while no significant metastatic lesions were detected in the EXO@CAT/US group (Figure 7C). These findings suggested that EXO@CAT/US treatment could significantly inhibit lung metastasis and show no apparent toxicity to normal tissues. In addition, the US-only group treatment showed no significant difference in antitumor efficacy and biosafety compared with PBS treatment (Figure S9), indicating that US treatment cause no significant destructive effects on tumors.

## Conclusion

In conclusion, we successfully extracted exosomes containing Cat and ACSL4, and further loaded TCPP to construct engineered exosomes termed EXO@CAT. This innovative approach combines endogenous proteins, which are prone to inactivation, with an exogenous sonosensitizer, allowing synergistic anticancer treatment of both ferroptosis and SDT with improved efficacy. The oxygen released by EXO@CAT not only facilitates its efficient penetration into the tumor tissue but also alleviates tumor hypoxia. This process generates a large amount of ^1^O_2_ under US irradiation, thereby triggering efficient ferroptosis in conjunction with ACSL4. *In vivo* studies demonstrated that after intravenous injection, EXO@CAT selectively accumulated in tumor tissues and achieved high penetration, resulting in desired anticancer effect under US stimulation. Therefore, EXO@CAT as a simple and effective formulation proposed in this study provides a promising new strategy for synergistic cancer treatment with SDT and ferroptosis, with significant implications for clinical applications.

## Supplementary Material

Supplementary figures.
