# Supplementary material for: Integrating oxygen-boosted sonodynamic therapy and ferroptosis via engineered exosomes for effective cancer treatment
Source: Theranostics. 2025 Jan 1;15(1):68–85. doi: 10.7150/thno.102977 (PMC11667237; doi:10.7150/thno.102977)
Supplement: Supplementary file 1 — Supplementary figures. [file thnov15p0068s1.pdf]

Supplementary data

Integrating oxygen-boosted sonodynamic therapy and ferroptosis via engineered exosomes for effective cancer treatment

Mingbo Wu[a,b,c,1], Zhanlin Zhang[c,1], Dong Li[b,1], Xiaomiao Ruan[a], Jingwen Yang[a], Siyi Chen[c], Xin Li[c], Wenwu Ling[a,*]

[a] *Department of Medical Ultrasound, West China Hospital, Sichuan University, Chengdu 610041, P.R. China*

[b] *Department of Oncology, The General Hospital of Western Theater Command, Chengdu610083, P.R. China*

[c] *School of Bioscience and Technology, Chengdu Medical College, Chengdu 610500, P.R. China*

[1]The authors contributed equally to this work.

*Corresponding author. West China Hospital, Sichuan University, No.37 Guoxue Alley, Wuhou District, Chengdu 610041, P.R. China. Phone: +8628-85422304

E-mail Address: lingwenwu@scu.edu.cn (W. Ling)

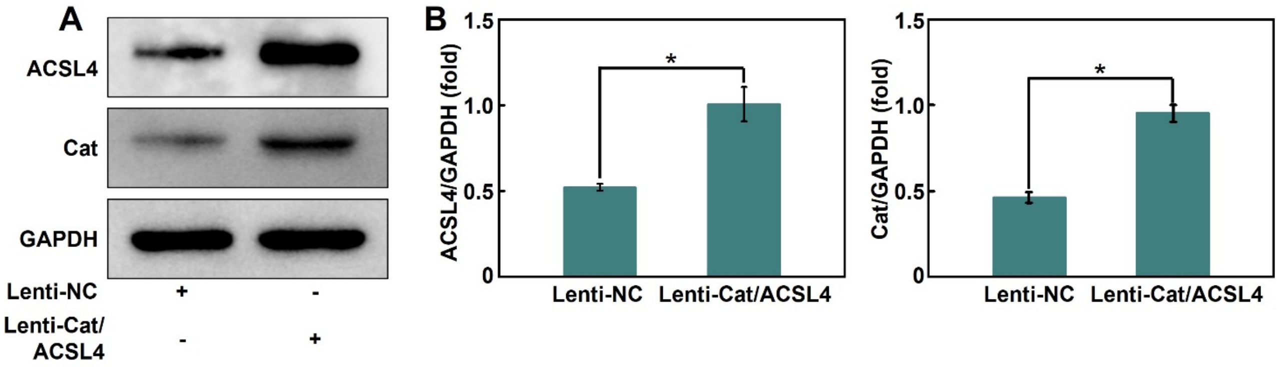

Figure S1. (A) Western blot and (B) quantitative analysis of ACSL4 and Cat expression in different transfected cells (n = 3, *: $p < 0.05$).

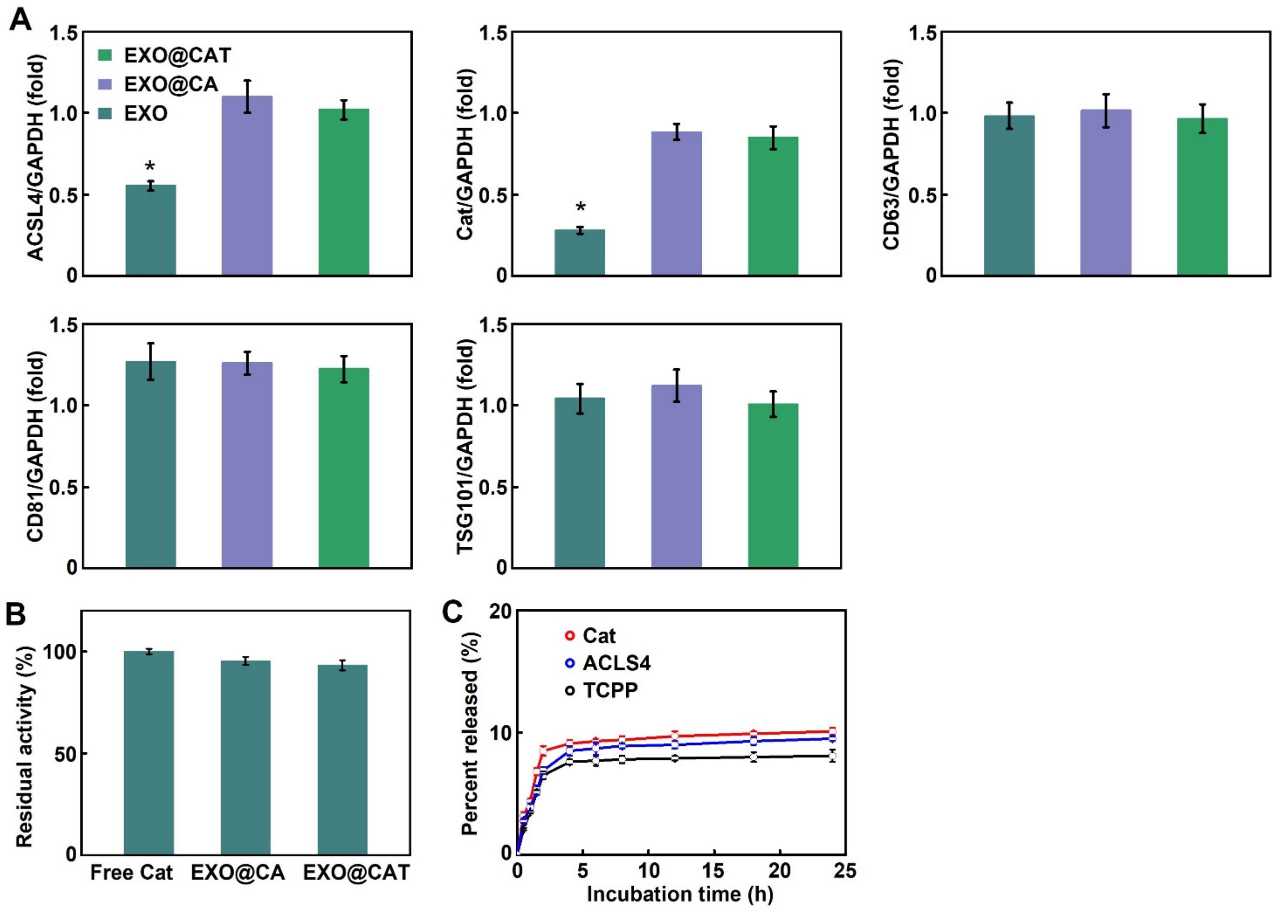

Figure S2. (A) Quantitative analysis of ACSL4, Cat, CD63, CD81, and TSG101expression in EXO NVs, EXO@CA NVs and EXO@CAT NVs (n = 3, *: $p < 0.05$ *vs* other treatments). (B) Cat activities of NVs at 37 °C and pH 7.4 compared with free Cat (n = 3). (C) Accumulated release of Cat, ACSL4, and TCPP from NVs in pH 7.4 buffers (n = 3).

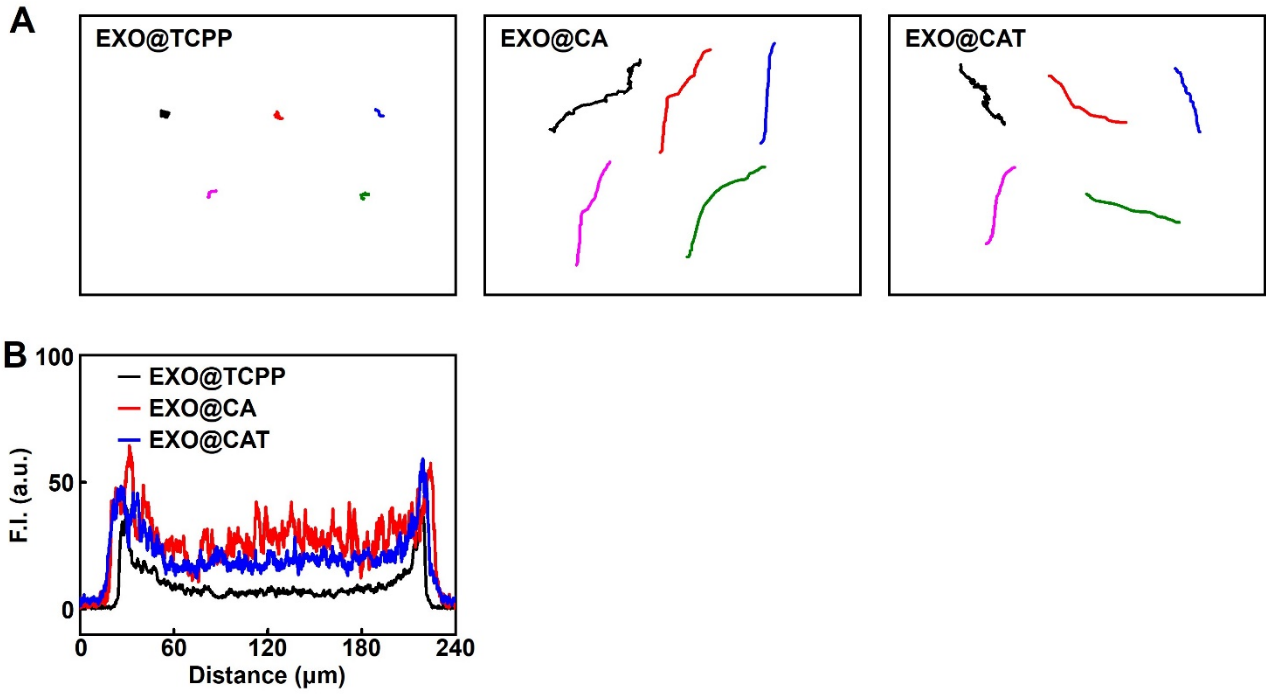

Figure S3. (A) Tracking trajectories of EXO@TCPP, EXO@CA and EXO@CAT NVs. (B) Corresponding fluorescence intensity profiles of *in vitro* penetration results in 3D tumor spheroids of EXO@TCPP NVs, EXO@CA NVs and EXO@CAT NVs (indicated by a yellow arrow in the Figure 2G).

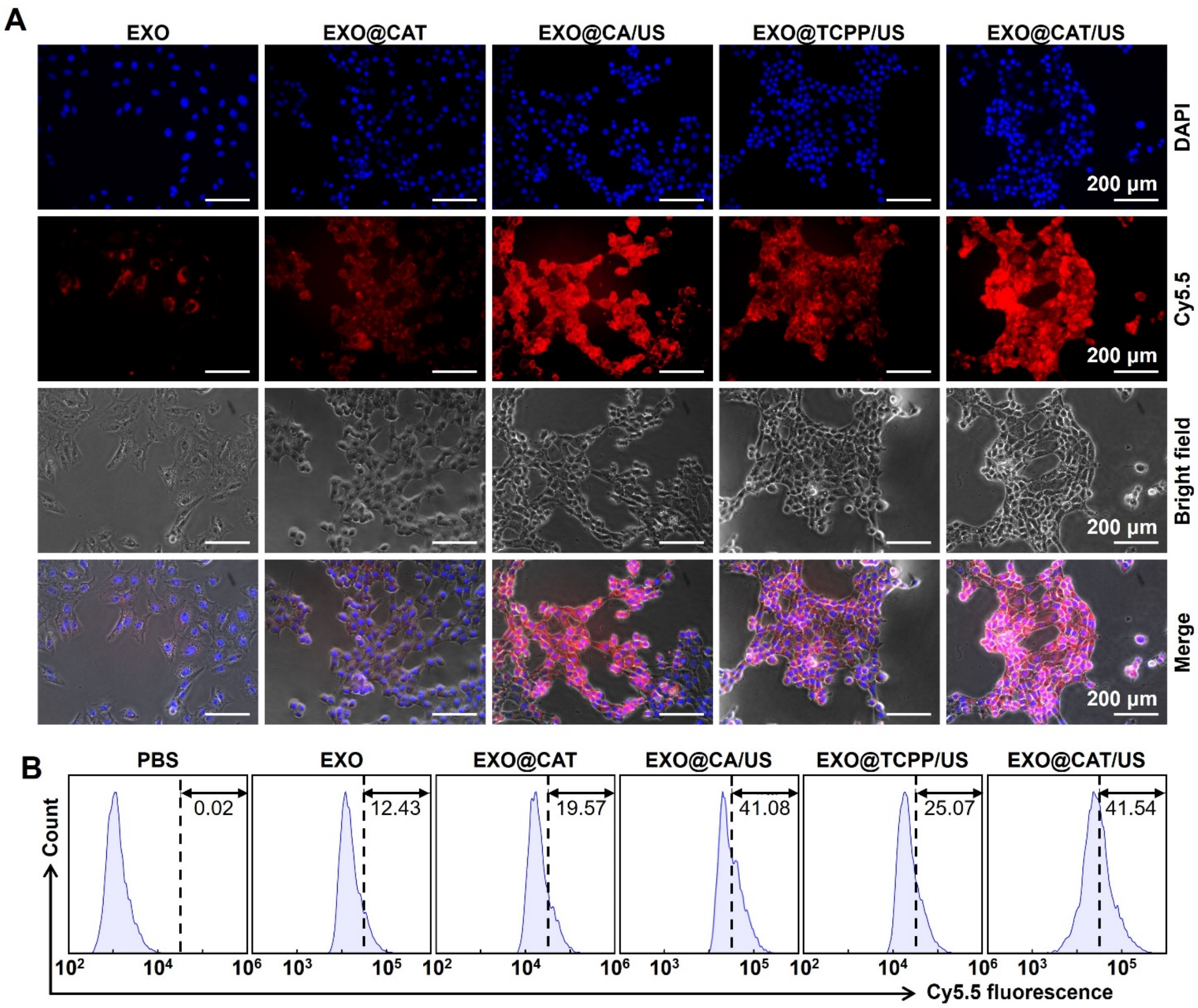

Figure S4. Cellular uptake of NVs. (A) CLSM images and (B) flow cytometry analysis of 4T1 cells after the treatment with Cy5.5 labeled NVs.

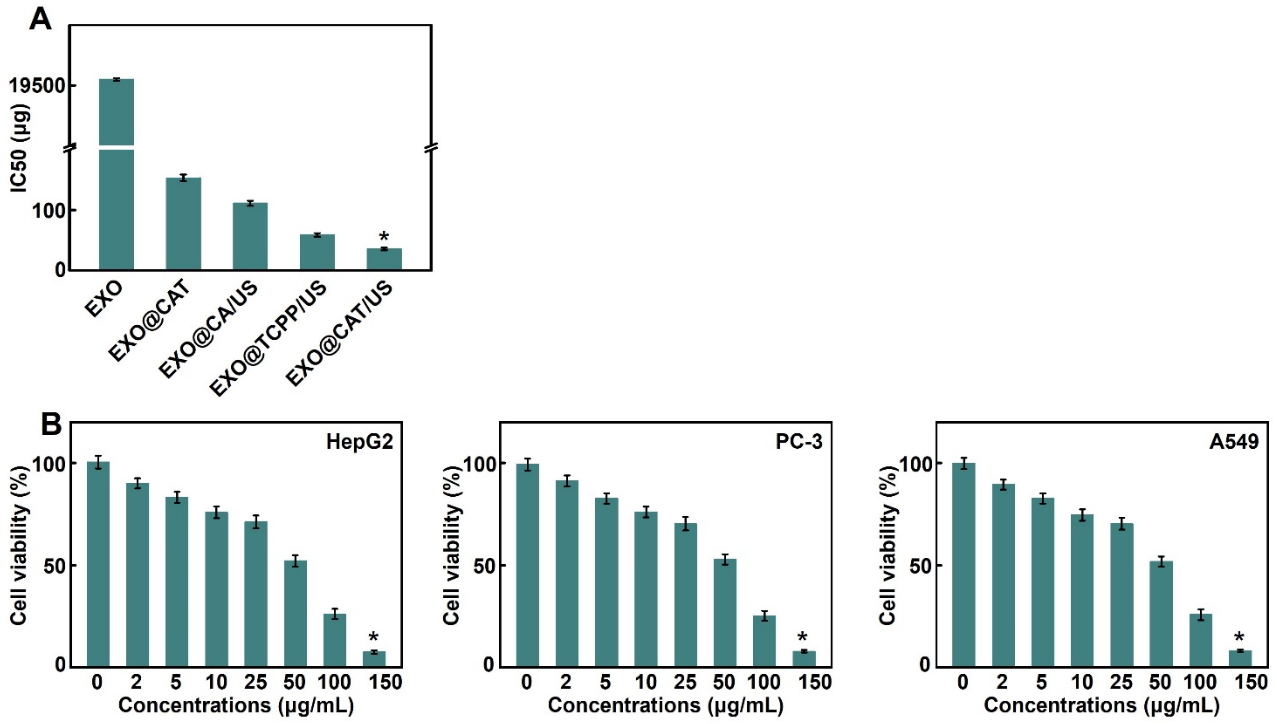

Figure S5. (A) IC50 summary of different treatments (*n* = 5, *: $p < 0.05$ *vs* other treatments). (B) HepG2, PC-3, and A549 cell viability after treatment with different concentrations of EXO@CAT NVs under ultrasonic irradiation (*n* = 5, *: $p < 0.05$ *vs* other treatments).

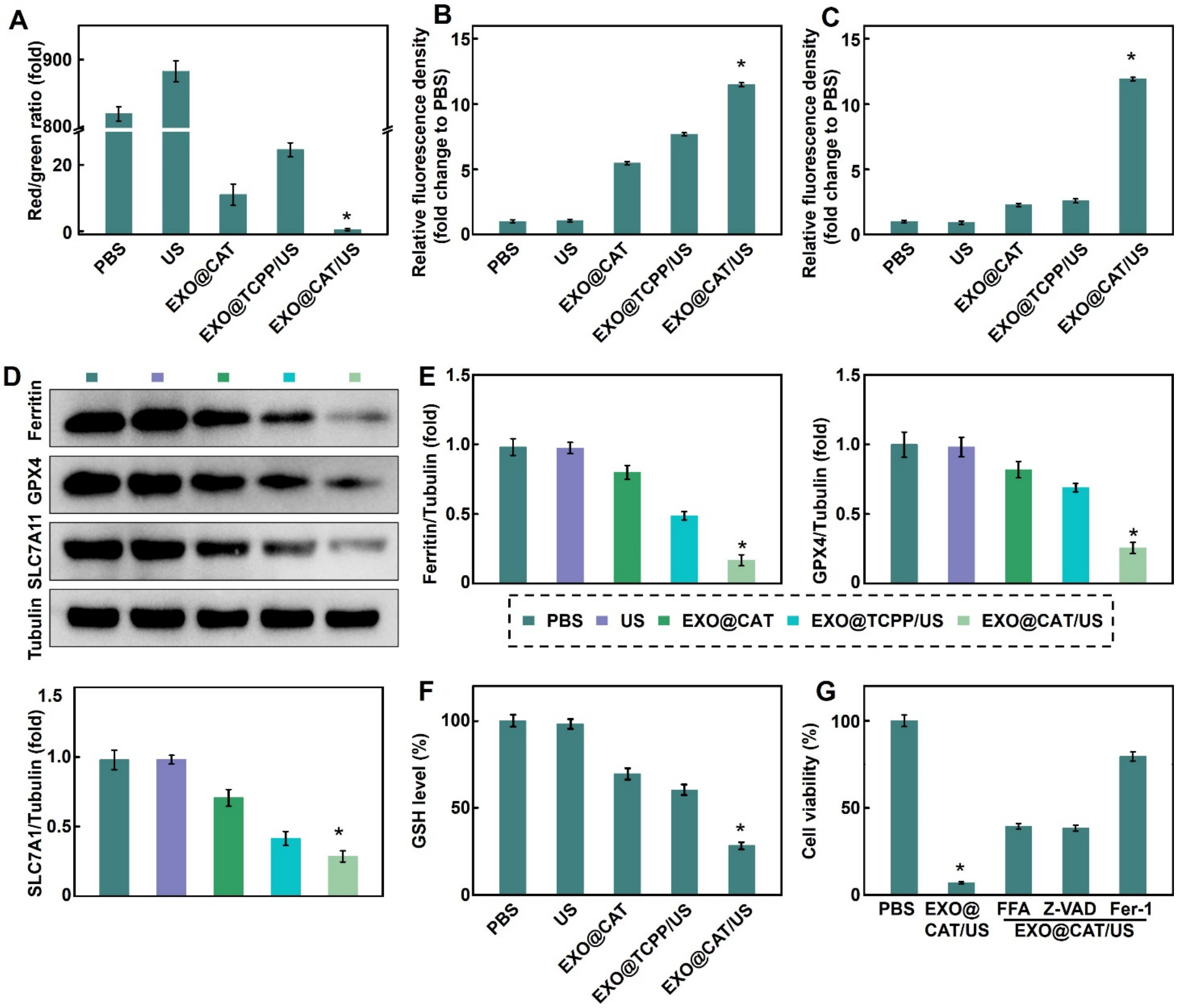

Figure S6. (A) Red/green ratio of JC-1, quantitative analysis of (B) Lip-ROS and (C) FerroOrange, (D) Western blot, (E) quantitative analysis of GPX4, Ferritin and SLC7A11 expression, (F) Intracellular GSH level in 4T1 cells after treatment with different NVs (*n* = 3, *: $p < 0.05$ *vs* other treatments). (G) Relative cell viability of 4T1 cells treated with EXO@CAT/US after the addition of FFA, Z-VAD-FMK, and Fer-1, respectively (*n* = 3, *: $p < 0.05$ *vs* other treatments)

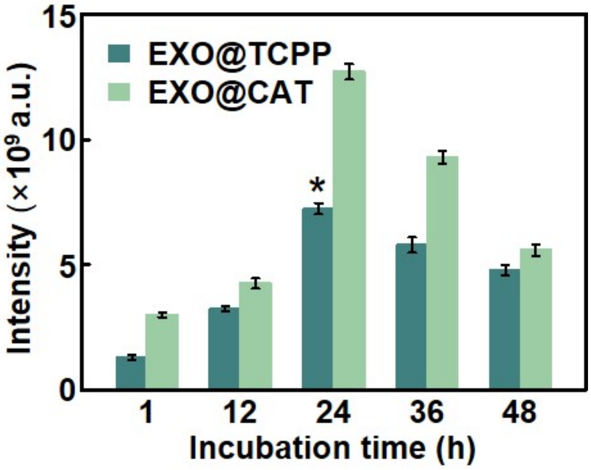

Figure S7. Quantitative analysis of fluorescence intensity after treated with EXO@TCPP and EXO@CAT (n = 3, *: p < 0.05 *vs* other treatments).

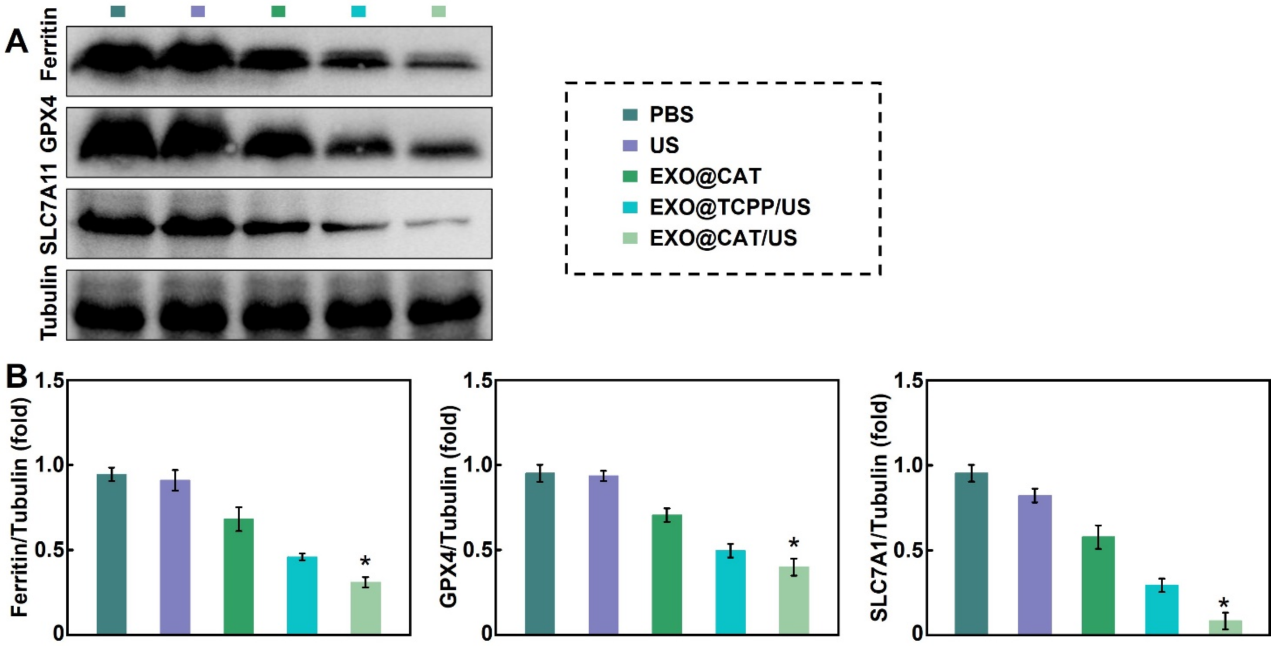

Figure S8. (A) Western blot, and (B) quantitative analysis of GPX4, Ferritin and SLC7A11 expression in tumor after treatment with different NVs (n = 3, *: p < 0.05 *vs* other treatments).

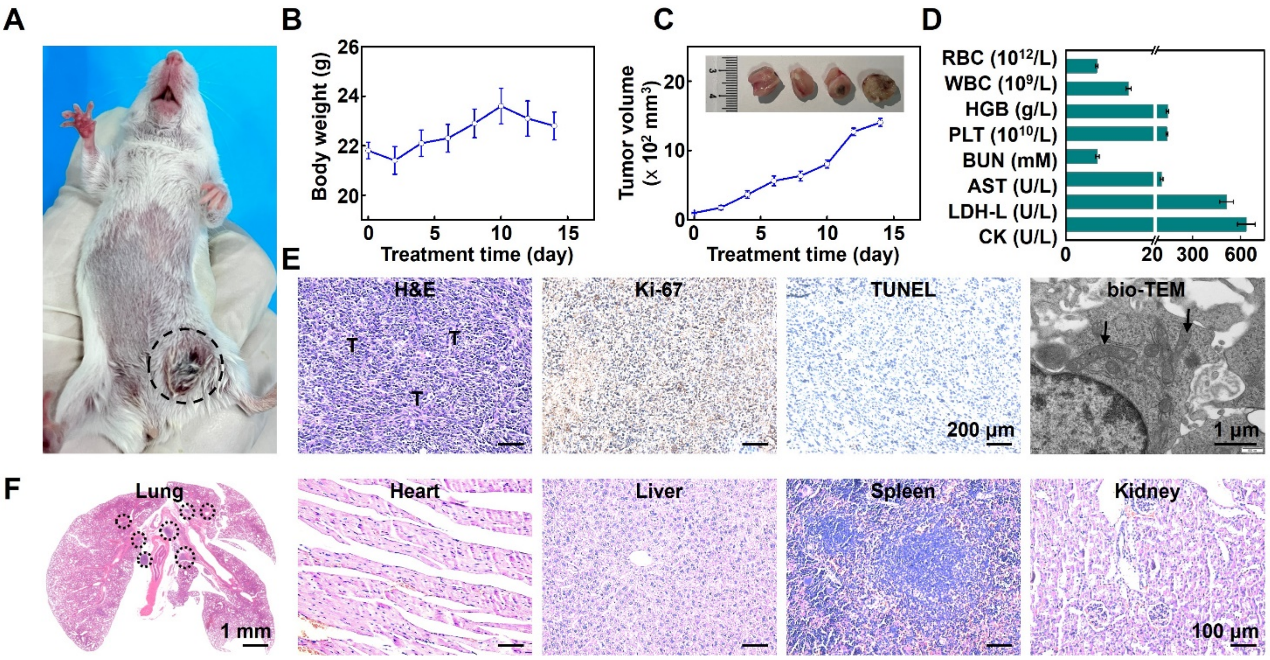

Figure S9. (A) *In vivo* representative pictures of 4T1 tumor-bearing mice (black circle represents the size and location of tumor). (B) Body weight changes of 4T1 tumor-bearing mice, (C) tumor growth curves, and the illustration is *in vitro* representative pictures of tumors. (D) Hematological and biochemical analyses. (E) H&E staining images ("T" represents tumor mass), Ki-67 staining images, TUNEL staining images, and bio-TEM in tumor sections after US-only group for 14 days, black arrow: normal mitochondria ($n = 4$). (F) H&E staining images of the heart, liver, spleen, lung and kidney after treatment with US for 14 days (black circle represents the tumor location of lung metastasis; $n = 3$).
